# *JANUS*: an open-source 3D printable perfusion bioreactor and numerical model-based design strategy for tissue engineering

**DOI:** 10.3389/fbioe.2023.1308096

**Published:** 2023-12-15

**Authors:** João Meneses, Sofia R. Fernandes, João C. Silva, Frederico Castelo Ferreira, Nuno Alves, Paula Pascoal-Faria

**Affiliations:** ^1^ Centre for Rapid and Sustainable Product Development, Polytechnic of Leiria, Marinha Grande, Portugal; ^2^ Instituto de Biofísica e Engenharia Biomédica, Faculdade de Ciências, Universidade de Lisboa, Lisboa, Portugal; ^3^ Department of Bioengineering and iBB—Institute of Bioengineering and Biosciences, Instituto Superior Técnico, Universidade de Lisboa, Lisboa, Portugal; ^4^ Associate Laboratory i4HB—Institute for Health and Bioeconomy, Instituto Superior Técnico, Universidade de Lisboa, Lisboa, Portugal; ^5^ Department of Mechanical Engineering, School of Technology and Management, Polytechnic of Leiria, Portugal; ^6^ Associate Laboratory for Advanced Production and Intelligent Systems (ARISE), Porto, Portugal; ^7^ Department of Mathematics, School of Technology and Management, Polytechnic of Leiria, Portugal

**Keywords:** bioreactor, modeling, design, capacitive-coupled, perfusion flow, multimodal stimulation, tissue engineering, finite element analysis

## Abstract

Bioreactors have been employed in tissue engineering to sustain longer and larger cell cultures, managing nutrient transfer and waste removal. Multiple designs have been developed, integrating sensor and stimulation technologies to improve cellular responses, such as proliferation and differentiation. The variability in bioreactor design, stimulation protocols, and cell culture conditions hampered comparison and replicability, possibly hiding biological evidence. This work proposes an open-source 3D printable design for a perfusion bioreactor and a numerical model-driven protocol development strategy for improved cell culture control. This bioreactor can simultaneously deliver capacitive-coupled electric field and fluid-induced shear stress stimulation, both stimulation systems were validated experimentally and in agreement with numerical predictions. A preliminary *in vitro* validation confirmed the suitability of the developed bioreactor to sustain viable cell cultures. The outputs from this strategy, physical and virtual, are openly available and can be used to improve comparison, replicability, and control in tissue engineering applications.

## 1 Introduction

Bioreactors for *in vitro* cultures are increasingly used in tissue engineering (TE) due to more complex mass transport requirements of growing tissues and also to allow controlled reproduction of specific cellular environments that can promote particular cellular processes by applying stimulation (e.g., mechanical, electrical). Understanding the microenvironment generated by bioreactors is crucial to predict cell survival and fate in TE strategies (Reina-Romo et al., 2019). *In silico* models have been successfully applied to guide the development and optimization steps towards a bioreactor design that favors the best cellular outcomes regarding seeding, proliferation, and differentiation (Spencer et al., 2013; Engel et al., 2021; Perier-Metz et al., 2021). Growing evidence report that multiple important environmental properties such as dissolved oxygen tension, glucose and lactate concentrations, and local pH value strongly impact stem cell fate. They should be closely monitored and controlled (Monfoulet et al., 2014; Beşkardeş et al., 2018; Seddiqi et al., 2020; Klein et al., 2021; Klein et al., 2022). However, simultaneous monitoring and controlling of specific cell microenvironments is difficult and time/resource-consuming. Predictions from computational studies help to determine the most relevant factors in the evolution and dynamics of biological systems (Geris et al., 2018), optimizing control and modulation of cell cultures in bioreactor systems in a low-cost virtual environment.

Many bioreactor designs have been proposed in TE to support better the proliferation and differentiation of several cell populations (Smith et al., 2018; Montorsi et al., 2022). Some bioreactors include a specific set of sensors to allow improved monitorization of the *in vitro* environmental conditions or actuator systems to apply different types of physical stimuli to promote cell differentiation (e.g., electric field, magnetic field, mechanical stress) (Lim et al., 2022). Even if proven effective, most of these designs are not shared open-source with proper fabrication and integration instructions, which hinders reproducibility and usage by the TE research community. The global massification of 3D printing technologies unlocks the opportunity to construct complex perfusion structures (Gensler et al., 2020) in suitable materials and techniques while retaining high customization freedom, reproducibility, and shareability at a low cost (Haleem et al., 2020).

In this work, we envision a strategy to iteratively design a perfusion bioreactor system based on model-driven decisions made on the microenvironment generated by each design hypothesis. These decisions aim to obtain cell culture conditions that promote a particular cell line’s high proliferation and differentiation rates [e.g., mesenchymal stem/stromal cells (Alvarez-Barreto et al., 2011; Bianconi et al., 2023), embryonic stem cells (Marolt et al., 2012), induced pluripotent stem cells (de Peppo et al., 2013)]. Perfusion technology was selected due to its natural double role in bioreactor systems allowing the renovation of the culture medium and, at the same time, applying fluid flow-induced wall shear stress stimuli to the tissue constructs, which has been shown to enhance MSC-mediated bone formation *in vitro* Wittkowske et al. (2016); Schröder et al. (2022); Yamada et al. (2022). For each microenvironment, finite element models were used to predict the volumetric distributions of fluid flow-induced shear stress and electric field magnitude in culture regions. The proposed perfusion bioreactor system can be fabricated with 3D printing technologies (e.g., fused filament fabrication, stereolithography) and accommodates the capability for simultaneous electrical and mechanical stimulation of four scaffolds in identical conditions. Perfusion technology was selected due to its natural double role in bioreactor systems allowing the renovation of the culture medium and, at the same time, applying fluid flow-induced wall shear stress. The journey pursued to get the proposed bioreactor design is presented, from early conceptualization to the current version, following a multiple trial and error approach. The strategy of early integrating numerical models of the bioreactor-generated microenvironment into the design phase allows trying different stimulation protocols and geometric options before fabrication, saving time and reducing operational costs. After fabrication, we experimentally validated the bioreactor system outputs against their numerical model outputs to increase the confidence in the developed strategy, obtaining a digital twin of the experimental setup capable of improving environmental control of cell cultures and, at the same time, capable of providing a framework to study the cellular effects of the applied stimuli. The complete developed solution, including all bioreactors’ components and numerical models, was named JANUS. This Roman inspiration describes a duality of physical and virtual representations. JANUS is available in an online repository (https://doi.org/10.5281/zenodo.7695700, released under an open-source Attribution-ShareAlike 4.0 International license). JANUS approach can be potentially applied to any cell culture study in the context of TE without any loss of applicability. Nevertheless, to establish design goals and allow *in vitro* validation, we directed the current development to bone tissue engineering (BTE) using a specific bone cell line since it constitutes an active research topic and a necessity to progress the understanding in this TE subarea (Sladkova and De Peppo, 2014). The presented bioreactor development was motivated by successive approaches to deliver adequate *in vitro* mechanical and electrical stimulation conditions to bone cells seeded on 3D-printed porous PCL scaffolds, in order to generate an osteogenic stimulation protocol that may mimic the native human bone microenvironment, and consequently, improve the regenerative outcomes.

## 2 Materials and methods

**FIGURE 1:**
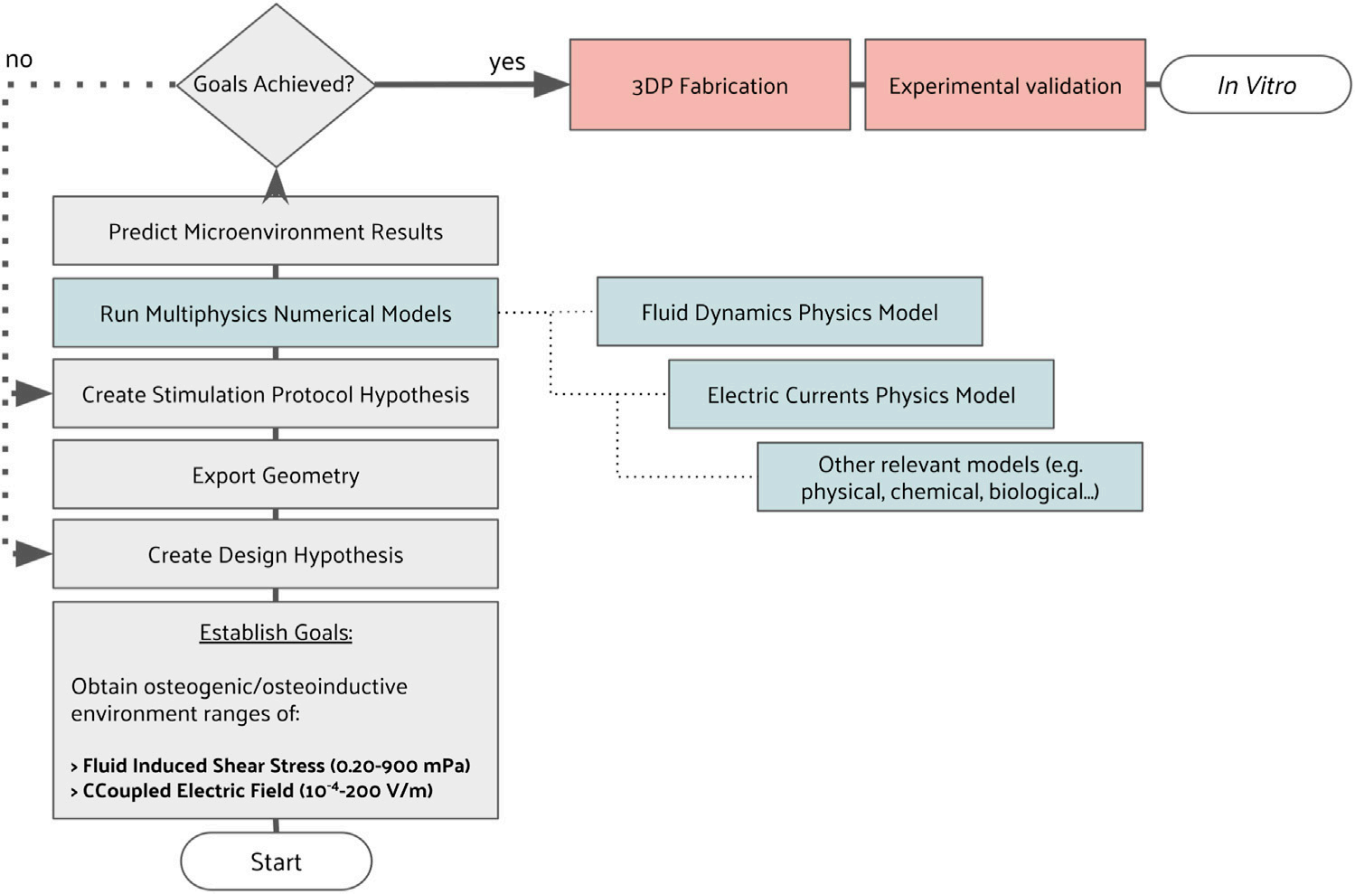
Decision tree used to iterate between CAD design and stimulation protocol hypotheses until FEM predictions matched an intended microenvironment.

A bottom-top approach to bioreactor design was guided by finite-element method (FEM)-based models. The decision tree applied for the bioreactor development is described in this section in a step-by-step manner (Figure 1). A cyclic iteration between CAD design and FEM predictions is conducted until the established microenvironment for that particular design is achieved. With this approach, we aim to reach a design that, after fabrication, can operate as numerically predicted. Here, we report the fundamentals of every development step, forwarding the reader to the Supplementary Materials, where a full description is written along with the complete roadmap of this bioreactor development (Supplementary Figure S1). To facilitate reproducibility, all systems developed in this work have only considered commercially available components or custom-made 3D printing parts.

### 2.1 Defining the bioreactor system development goals

The starting point for this design was a previously developed bioreactor concept (Meneses et al., 2020; Meneses et al., 2022b). This design was progressively modified to integrate multiple actuators and sensors while preserving the ability to be entirely 3D printable (Supplementary Table S1). Culture medium volume usage was minimized, and sensor probes were included for online monitoring (pH, dissolved oxygen, temperature).

Perfusion bioreactors allow providing cell-seeded scaffolds with homogeneous nutritional supply while removing waste products effectively, favoring appropriate cell metabolic activity (Gaspar et al., 2012; Shakeel et al., 2013). Also, the induced fluid flow shear stress can simultaneously act as mechanical stimuli to promote improved responses in some cellular types (e.g., bone cells). Due to these two attributes, perfusion technology was selected for this development to ensure proper osteogenic conditions. We chose a wall shear stress range in agreement with those previously observed to produce osteoinductive effects for mesenchymal stem/stromal cells. We refer to the osteoinductive ranges of 1.47–24 mPa (Vetsch et al., 2017) and 0.20–13.35 mPa (Yamada et al., 2021). We emphasize that the resultant wall shear stress range is a product of the bioreactor-generated fluid flow characteristics, being also influenced by the scaffold properties, including its geometry and surface topology.

The other technical decision was to select adequate electric field stimulation technology for *in vitro* cultures. Evidence of electric field technologies to support osteogenic processes has built up over the last decades and is extensively reviewed by Nicksic et al. (2022). We decided on capacitive coupled (CCoupled) systems since these ensure the delivery of a pure electric field stimulation without faradaic byproducts or an accompanying magnetic field. CCoupled electrodes were considered, fabricated from indium tin oxide coated polyethylene terephthalate (ITO PET) films (33 × 18 mm), and a 175 µm-thick polyester film coated with indium tin oxide (60 Ω/sq) that was glued with polydimethylsiloxane (PDMS) to a 3D printed structure. Regarding CCoupled stimulation, the system will be designed to deliver an electric field magnitude with a wide range, considering the values previously reported of ×1.0 10^−5^ to 1.3 × 10^3^ V m^−1^ (Korenstein et al., 1984; Fitzsimmons et al., 1986), using a frequency of 60 kHz as applied by other previous CCoupled works addressing bone regeneration (Brighton et al., 1992; Stephan et al., 2020).

### 2.2 Creating geometrical design hypotheses

**FIGURE 2:**
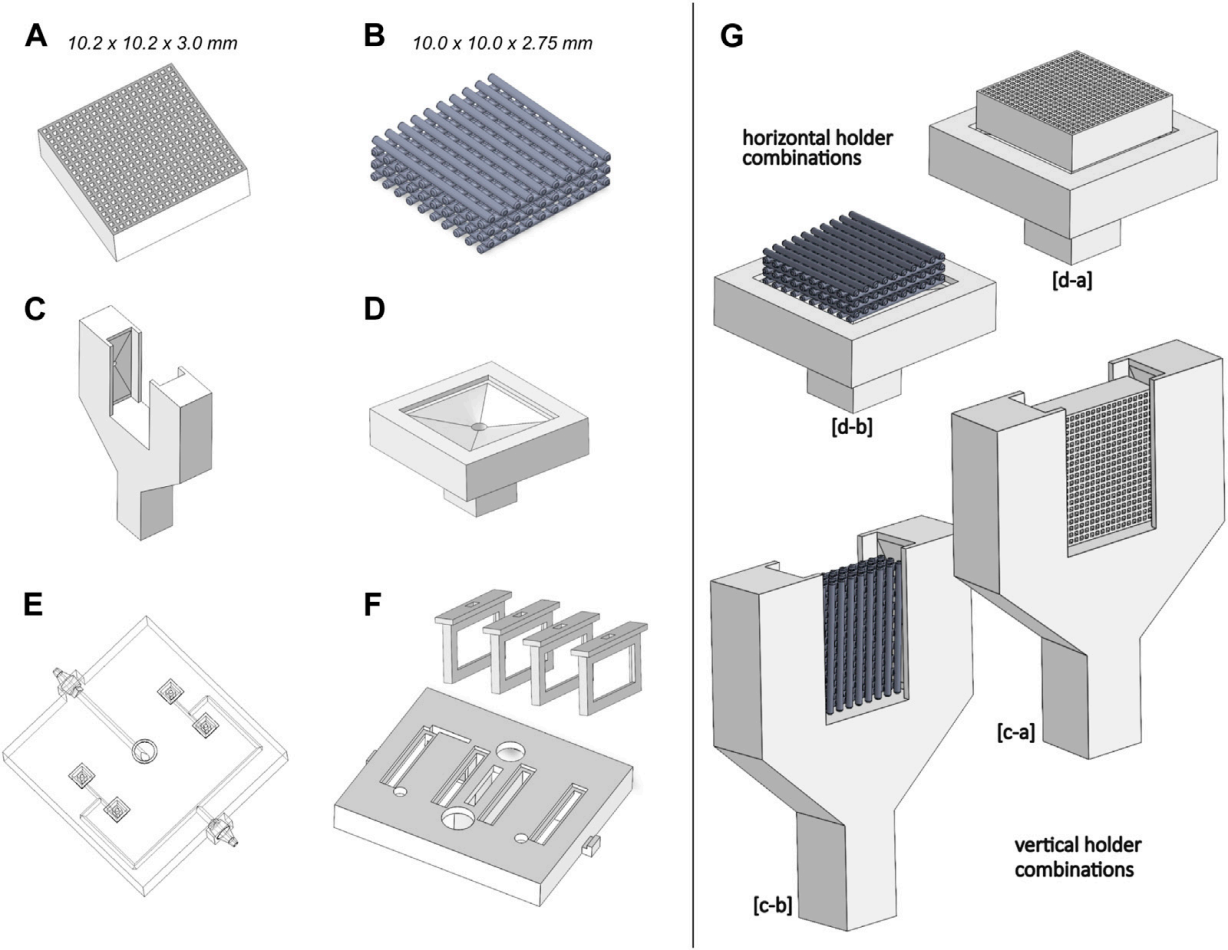
Geometrical design hypotheses considered. **(A)** Honeycomb scaffold structure; **(B)** Orthogonal scaffold structure; **(C)** Vertical scaffold holder; **(D)** Horizontal scaffold holder; **(E)** Outlet channel network; **(F)** Support for CCoupled electrodes; **(G)** Combinations of scaffold and holder considered for simulations.

All bioreactor and scaffold parts were designed with SOLIDWORKS (2018 Student Edition, Dassault Sistèmes), a parametric computer-aided design (CAD) software. Two scaffold geometries were selected from the literature as an example to apply the described development methodology (Figures 2A, B). Our selection criteria were to consider scaffold geometries actively used in bone tissue engineering research, capable of matching the mechanical properties of cortical or trabecular bone formations to some extent.

The first scaffold geometry selected is from Hayashi et al. (2019); Hayashi et al. (2020); Hayashi et al. (2022); Shibahara et al. (2022), consisting of a honeycomb structure scaffold for bone regeneration, made from carbonate apatite to resemble natural human bone mineral composition. Their studies tested different macropore and micropore volumes, demonstrating that high interconnectivity and uniformity of channels enable scaffolds to maintain high mechanical properties and osteogenic ability while being suitable to be applied as implants for weight-bearing areas (Hayashi et al., 2019; Hayashi et al., 2020). This honeycomb scaffold structure was produced by extrusion molding, with a reported Young’s moduli of 23 GPa (Hayashi et al., 2020), higher than the usual Young’s moduli value for cortical (18–21 GPa) or trabecular (10–15 GPa) bone formations (Morgan et al., 2018), but with the potential to be tailored by porous structures design to mimic the mechanical properties of bone structures. The CAD geometry of the honeycomb structure scaffold was considered with an external envelope volume of 10.2 × 10.2 × 3.0 mm, a truss size of 250 µm and a macropore size of 300 µm.

The second scaffold geometry selected is a regular orthogonal scaffold that, when produced from ceramic materials, such as biphasic calcium phosphate (Touri et al., 2018) or lithium-calcium-silicate crystal (Chen et al., 2019), has reported properties similar to native bone minerals. The CAD geometry of the orthogonal scaffold presents an external envelope volume of 10.0 × 10.0 × 2.75 mm while maintaining the geometrical relations and dimensions reported by Touri et al. (2018), with a filament diameter and pore size of 500 µm. Due to typical 3D printing fabrication constraints and to ease numerical models, a filament superposition of 10% and round fillets of 0.02 mm diameter were added to each orthogonal intersection.

In addition to the scaffold design hypothesis, two versions of scaffold holders were conceived to provide support in different positions (vertical vs. horizontal), also incorporating different outlet flow channels (Figures 2C, D). The reason for this holder hypothesis was to provide different fluid flow characteristics (one bottom outlet vs. two lateral outlets), and also, since the CCoupled system is mounted laterally, the created holder hypothesis allows testing two electric stimulation configurations. To connect each of the bioreactor’s four scaffold holders to the outlet peristaltic tube, a channel network (Figure 2E) was designed to guarantee that, at every channel split, the sum of section areas of the child branches is equal to the section area of the parent branch, condition necessary to minimize flow velocities loss, since it divides the outlet flow equally among all supported scaffolds. Regarding the CCoupled system, since stimulation amplitude, waveform, and duration are the most determinant parameters for the CCoupled effects, all geometrical variations of electrode number, position, or size were not considered in this work, being described elsewhere (Tandon et al., 2008). Thus, the delivery range of the electric field in this work was varied only by changing the input waveform of the stimulation protocol. The electrodes for the CCoupled system were placed in parallel positions and equidistant from the scaffold center, 22 mm apart, in such a way that each electrode pair will stimulate two side-by-side scaffolds (Figure 2F).

The decision to have four scaffolds per bioreactor was to increase statistical power in the experimental condition, being a common practice in TE to replicate the same condition in a high number of samples (*N* > 3) (Pollard et al., 2019; Silva et al., 2020). To avoid unnecessary simulations and decrease computational effort, the proposed work was separated into two blocks: 1) obtain the outlet channel network design that minimizes the fluid flow velocity loss; 2) all combinations of scaffolds and holders designs were considered, resulting in four models that were analyzed with fluid flow and electric field simulation studies. The combination of geometries and protocol which predictably results in the closest conditions to an osteogenic microenvironment were selected for fabrication and validation.

### 2.3 Setting simulation input parameters

Each protocol was selected considering the maximum output of the available lab equipment (electric signal source and peristaltic pump), guaranteeing that the highest magnitudes possible for the available lab equipment were obtained for each stimulation condition at the cell culture chamber. Once determined the bioreactor-generated microenvironments for the maximum equipment’s output, we established our working baseline: the peristaltic pump flow rate was set to the maximum continuous outlet flow of 50 mL min^−1^, mounted with a 2.79 mm internal diameter peristaltic tube (0.328 m/s at the peristaltic tube end); the CCoupled input waveform generator was set to its maximum amplitude of 10 V_p-p_ for a sinusoidal wave with 60 kHz. Both stimulation protocol parameters were considered as inputs for the developed numerical models.

### 2.4 Multiphysics numerical simulations

Each designed part was exported to the STEP file format, allowing it to be imported and post-processed by COMSOL Multiphysics finite-element analysis (FEA) software (version 5.2a, www.comsol.com). Meshing was performed with the physics-controlled mesh and fine options. These options translated to meshes made from free tetrahedral elements with an average element quality of 0.68. Further mesh characterization data is available in the COMSOL reports, downloadable in an online repository (https://doi.org/10.5281/zenodo.7695700). Meshes were constructed with a high number of nodes to guarantee mesh-independent results (computable by the processor AMD Ryzen 7 5700G 8-Core 3.8 GHz c/Turbo 4.6 GHz 20 MB SktAM4). Two COMSOL physics interfaces were applied for each stimulation mode. For fluid flow shear stress calculations, computer fluid dynamics (CFD) stationary studies were conducted with the single phase Laminar Flow physics interface, which solves the Navier-Stokes equations for the conservation of momentum and the continuity equation for the conservation of mass. The cell culture medium was considered as an incompressible Newtonian fluid, with viscous material properties similar to water, as applied by Hidalgo-Bastida et al. (2012) and in our previous works (Meneses et al., 2020; Meneses et al., 2022b). Outlet channel network flow models were imposed with an outlet velocity boundary condition of 0.328 m s^−1^ applied to the peristaltic tube connector, and a standard atmosphere pressure (1.01 × 10^5^ Pa) inlet boundary condition applied to the scaffold holder connector channel. Flow models for scaffold and holder combinations were enclosed by a cylindrical volume with a radius of 20 mm and a height of 30 mm to represent a part of the culture medium domain inside the bioreactor chamber. The atmospheric pressure inlet boundary condition was considered in all surrounding cylindrical surfaces. In contrast, a single outlet boundary condition was added to the exit surface of the scaffold holder channel (designed with equal dimensions for both holder options). The scaffold holder outlet boundary condition was defined with the average velocity magnitude value (0.120 m s^−1^) obtained from the first outlet channel network model solution.

CCoupled electric field stationary calculations were performed with the Electric Currents physics interface, solving a current conservation equation based on Ohm’s law using the scalar electric potential as the dependent variable, assuming the quasistatic approximation as applied by Budde et al. (2019) and in our previous studies (Meneses et al., 2022a; Fernandes et al., 2022). Electric potential boundary conditions were added to the outer surfaces of electrodes, 5 V to the active electrode (corresponding to 10 V_p-p_), 0 V to the other electrode (ground). A frequency domain study was conducted at 60 kHz.

Material properties of each domain were set according to Table 1 for simulated protocols. FEA was post-processed with COMSOL for each hypothesis, considering the envelope volume surrounded by the selected cell culture scaffold as the region of interest (ROI).

**TABLE 1:** Material properties for numerical model domains.

Material	Properties
Osteogenic Culture Medium (37°C)	Electric conductivity: 1.5 S m^−1^
	Relative permittivity: 80.1
	Kinematic viscosity: 6.89 × 10^−4^ Pa.s
	Density: 9.94 × 10^2^ kgm^−3^ Gabetti et al. (2022)
Electrode - ITO part	Electric Conductivity: 1.0 × 10^6^ S m^−1^
	Relative permittivity: 1 MIT (2023)
Electrode - PET part	Electric Conductivity: 1.0 × 10^−21^ S m^−1^
	Relative permittivity: 3 MIT (2023)
C8 (PLA composite) - bioreactor parts	Electric Conductivity: 1.0 × 10^−21^ S m^−1^
	Relative permittivity: 2.7 Hegde et al. (2015)
PCL - scaffold	Electric Conductivity: 1.0 × 10^−13^ S m^−1^
	Relative permittivity: 3.2 Hegde et al. (2015)

COMSOL reports were generated for each numerical study and are available for download in an open-source repository (https://doi.org/10.5281/zenodo.7695700), containing a detailed description of all the parameters required to replicate the numerical research, following a documenting standard for TE stimulation studies proposed by Budde et al. (2019). The volumetric distribution of Reynolds number, fluid-induced shear stress, fluid flow overall velocity, and axial component velocity magnitude was analyzed for CFD models. Fluid-induced shear stress was calculated from COMSOL shear rate and water dynamic viscosity at 37°C (approximation valid for Newtonian fluids (Wilson, 2018)). For CCoupled models, the volumetric distribution of electric field magnitude and integration of the resultant electric current was analyzed.

### 2.5 Fabrication of the bioreactor, scaffold, and supporting systems

All 3D printable parts selected for production from the design hypothesis were fabricated with proprietary C8 material (3D4Makers, Netherlands) and printed with an Ender 3 S1 Pro 3D FFF printer (Creality, China). C8 material was previously subjected to *in vitro* cytotoxicity tests, showing to be compatible with cell culture experiments Meneses et al. (2020). The printer specifications were set accordingly with the C8 manufacturer datasheet (printing temperature: 210°C, bed temperature: 50°C, maximum printing speed: 35 mm/s). Connectors and other support parts were fixed and isolated with PDMS (Sylgard 184 Silicone Elastomer Kit, applied in a 10:1 (w/w) ratio of base to curing agent) and left to dry overnight. The perfusion system was developed based on commercially available peristaltic pumps (Supplementary Figures S2–S4). Custom sensor circuits, firmware, and software interfaces (Supplementary Figures S5–S7) were developed to communicate commands via Bluetooth protocol to the bioreactor (Supplementary Table S2). The scaffold geometry predicted to ensure the most optimal microenvironment was selected and 3D printed with polycaprolactone (PCL) material and evaluated for structural/morphological properties by micro-computed tomography using a SkyScan 1174TM (Brucker, Kontich, Belgium). Blueprints with all fabrication instructions and required source files are described in the Supplementary Materials and available for download in an open-source repository (https://doi.org/10.5281/zenodo.7695700).

### 2.6 Pre-culture validation

All 3D-printed parts and electronic systems were tested individually to guarantee proper functioning. A complete description of each validation test may be found in the Supplementary Materials, which include watertight tests, electronics, and communication operational tests. Individual sensor outputs were validated against golden standard devices or standard solutions. Their predictions were compared with experimental measurements of the fluid flow velocity and total electric current that passes through the CCoupled system to validate the fabricated designs and correspondent numerical models. Both perfusion and CCoupled systems were tested independently of one another. Notably, the CCoupled system designed to be mounted on the top of the developed bioreactor perfusion chamber was tested in a specifically designed support that matches its final configuration. ITO PET capacitive electrodes were mounted 10 mm apart (see Figure 6), separated by culture medium.

### 2.7 Cell viability and metabolic activity validation after bioreactor culture

A preliminary cell culture validation was performed with the proposed bioreactor system using PCL scaffolds seeded with Human osteoblast-like MG-63 cells (ATCC®CRL-1427™) under fluid flow static conditions (without perfusion), with and without the application of the previously defined CCoupled stimulation protocol (sinewave amplitude from 5 V to −5 V, 60 kHz, 1 h per day). A complete description of the applied cell culture process is available in the Supplementary Materials. Briefly, the entire bioreactor system was sterilized by means of ethanol 70% washing followed by 1% v/v antibiotic-antimycotic (Gibco™) solution (prepared in Phosphate Buffered Saline (PBS)) washing (all the bioreactor parts and also on tubing through perfusion using the peristaltic pump) and by ultraviolet light exposure for 2 h. Then, human MG-63 osteoblasts were seeded (200,000 cells/scaffold) on the 3D-printed PCL scaffolds and cultured for 12 days in static conditions to promote the population of the whole scaffold structure. Then, the cell-seeded scaffolds were transferred to the fabricated bioreactors and cultured for 48 h (2 days inside the bioreactor placed inside an incubator at 37°C and 5% CO_2_). Three experimental groups were created to evaluate cell viability and metabolic activity. A control group comprised cell-seeded scaffolds cultured on a well-plate static culture. Two test groups were formed of cell-seeded scaffolds cultured in the proposed bioreactor static culture with/without electric stimulation (*N* = 4). Perfusion conditions were not considered for preliminary tests to allow better comparison with cell plate standard culture and with reported experimental studies of electromagnetic stimulation of Human osteoblast-like MG-63 cells, also performed under static medium conditions. Cell viability was assessed with a LIVE/DEAD staining (Life Technologies), and the metabolic activity was evaluated via the Alamar Blue assay (Thermo Fisher Scientific), both protocols are available in Supplementary Materials.

### 2.8 Statistical analysis

Statistical data analysis was performed by one-way ANOVA, followed by a Tukey post-hoc test using the GraphPad Prism 7.0 software (GraphPad, San Diego, CA, United States). Data were considered statistically significant when the *p*-values obtained were less than 0.05 (95% confidence intervals, *p* < 0.05).

## 3 Results

This section presents results from numerical models, starting from the predicted generated microenvironment for each bioreactor, scaffold, and holder design hypothesis considered. Then, after selecting the design that predictably generates the most osteogenic microenvironment, results from experimental validation using the developed systems are presented and compared with predictions from their numerical models. Finally, *in vitro* preliminary cell culture validation results are described regarding cellular viability and metabolic activity.

### 3.1 Predicted bioreactor microenvironments

**FIGURE 3:**
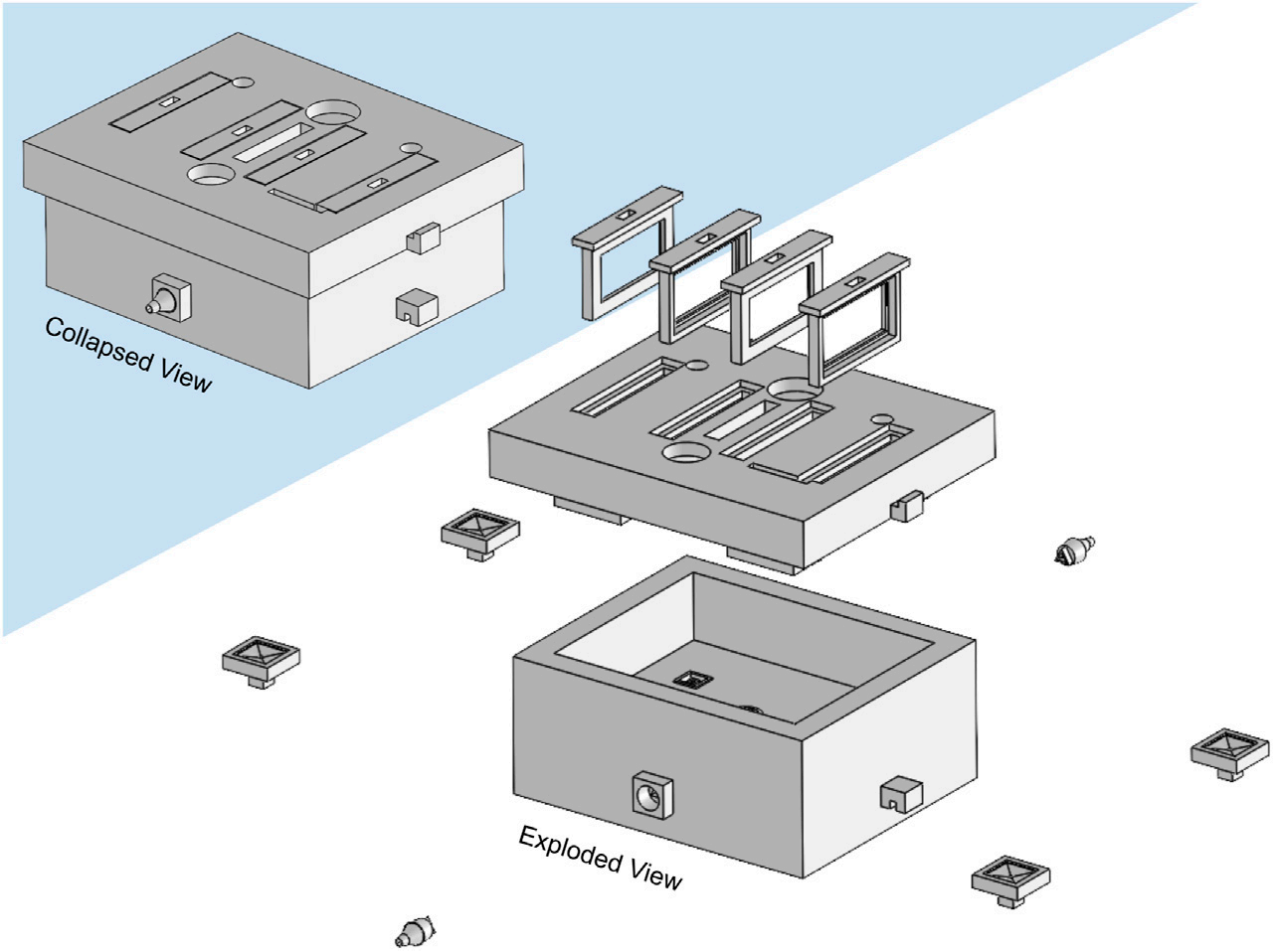
Illustration of the developed bioreactor and its components. Left upper corner: assembled collapsed view. Right bottom corner: exploded view. Only the horizontal scaffold holders option is represented.

The developed bioreactor design (Figure 3) minimizes the culture medium volume to 45 cm^3^, a decrease of 91% from our previous bioreactor version (see Supplementary Materials), reducing significantly culture media-related costs. This volume reduction also impacted the channel network design. From CFD analysis, we estimated a surface average velocity magnitude of 0.120 m s^−1^ at each scaffold holder connector channel, corresponding to an outlet velocity of 0.328 m s^−1^ for the maximum peristaltic pump flow rate of 50 mL min^−1^ (hose connector section area of 0.0254 cm^2^). The CFD velocity profiles of all four scaffold holder connector channels were predicted to be equal, according to the construction design strategy adopted and explained in the methods section.

**FIGURE 4:**
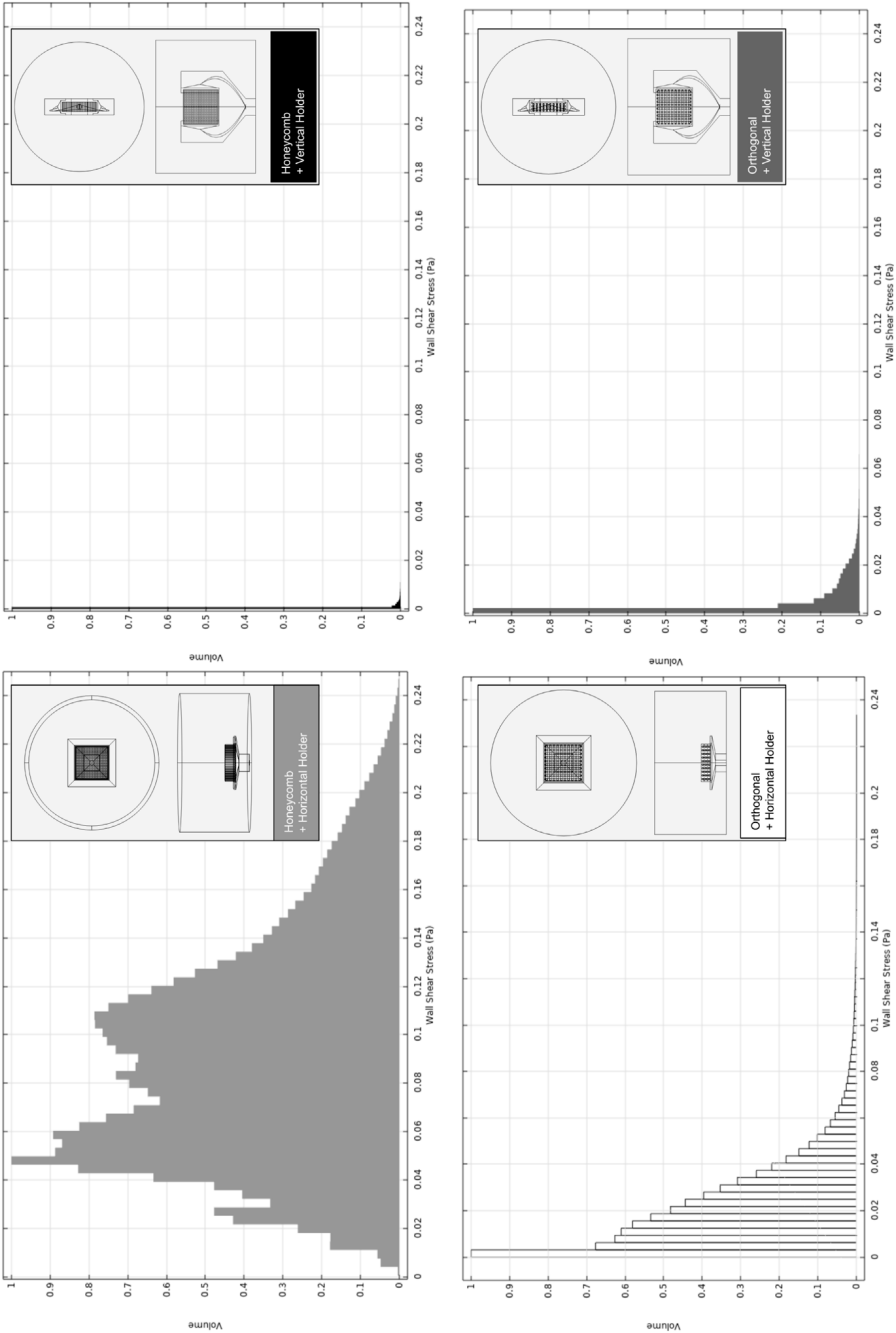
Relative volumetric distribution of the fluid-induced shear stress (in units of Pa) for all combinations of selected scaffolds and holders, predicted by the CFD numerical model at the culture ROI. The maximum peristaltic pump rate of 50 mL min^−1^ was considered at the outlet. The volume axis refers to the number of node occurrences of the correspondent shear stress in the ROI, normalized to its peak value.

The two holders and scaffold designs were combined into four design hypotheses (a-c, a-d, b-c, and b-d are all created combinations as shown in Figure 2). For the outlet’s perfusion velocity (at maximum peristaltic pump flow rate), the Reynolds number predicted was within the laminar flow limits (<0.1) (LaNasa and Loy Upp, 2014) for all design combinations. Histograms for the volume distribution of the fluid-induced shear stress are presented in Figure 4 (correspondent velocities magnitude are available in Supplementary Figure S8).

The holder at the horizontal position originates a broader shear stress spectrum for both selected scaffold geometries. Once we are using the maximum peristaltic pump flow rate, this means that the horizontal holder configuration is the only one that may allow us to tune the fluid-induced shear stress by decreasing/increasing the peristaltic pump flow rate debit if required. In Supplementary Figure S8A, the impact of changing the peristaltic pump flow rate is exemplified, an action that will allow fitting the fluid flow-induced shear stress to a recommended cellular range of mechanical stimulation. Even considering the outcomes of the considered maximum peristaltic pump flow rate, at cell culture ROI, if both scaffold designs were placed in the horizontal holder, both would be able to generate a microenvironment inside the reported osteogenic ranges, 0.0015–0.024 Pa (Vetsch et al., 2017), 0.00020–0.013 Pa (Yamada et al., 2021), (Figure 4; Supplementary Figure S8B). It is observable in the numerical predictions (Figure 4) for all considered scaffold and holder combined geometries that most of the volume fractions values occur within a range from 0 to 0.1 Pa. Remarkably, the horizontal holder and orthogonal scaffold combination show most of the predicted volume fractions in a range of 0–0.02 Pa, already inside the reported osteogenic ranges.

The horizontal holder was then selected since it provided optimal fluid-induced shear stress conditions for osteogenic effects, and the predicted volumetric distributions of the electric field for this holder and the established protocol were then compared (see section 2.4).

**FIGURE 5:**
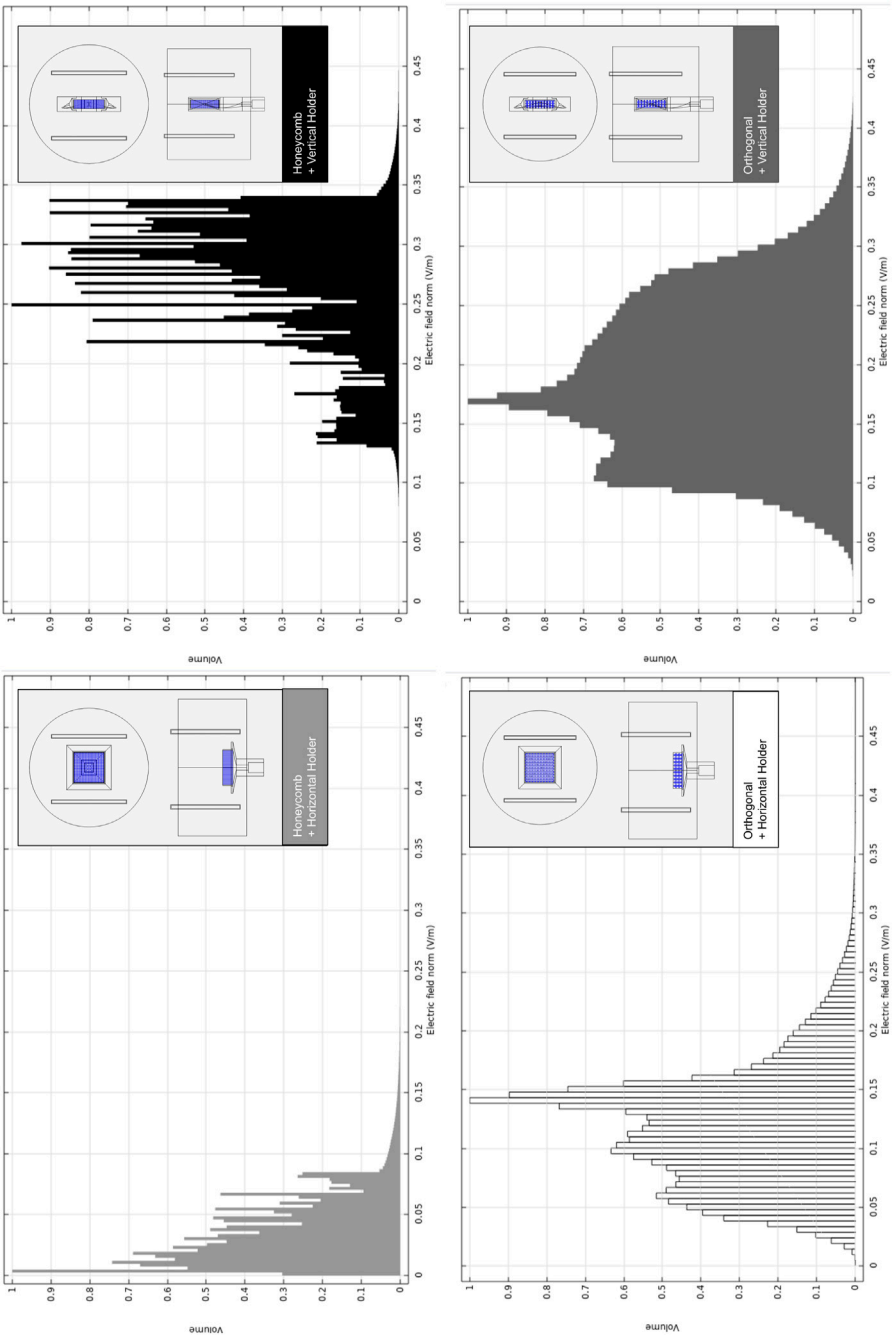
Relative volumetric distribution of the electric field magnitude (V m^−1^) for all combinations of scaffolds and holders when subjected to the CCoupled stimulation (sine wave, 60 kHz, 10 Vp-p). All data was obtained from frequency domain electric field numerical model predictions at the culture ROI. The volume axis refers to the number of node occurrences of the correspondent electric field magnitude in the ROI, normalized to its peak value.

When comparing both scaffold geometries placed upon the horizontal holder (Figure 5) for the same electric stimulation protocol, the orthogonal scaffold is the one with a higher electric field magnitude predicted at culture ROI, with an average value of 0.118 V m^−1^. In comparison, the honeycomb scaffold has an average electric field magnitude of 0.035 V m^−1^. The observed non-uniformity of the electric field follows previously reported studies Meneses et al. (2021). These prediction results are caused by the presence of a scaffold structure that introduces an obstacle to the flow of charges (Supplementary Figure S8B). The effect of the scaffold’s presence on the electric field in the surrounding culture medium is mainly determined by its geometry and by the difference in electrical conductivity of the scaffold materials and surrounding culture medium. Combining the horizontal holder with the orthogonal scaffold thus allows for a broader range of multimodal stimulation (simultaneous fluid-induced shear stress and electric field). For this reason, this was the hypothesis selected for fabrication and subsequent validation.

### 3.2 Fabrication and pre-culture validation

Bioreactor parts, selected scaffold, and holder structures were 3D printed as described in the methods section. Systems electronics were assembled, and probes were inserted into their established positions inside the bioreactor (see Supplementary Figures S2–S9). Pre-culture tests were performed as described in the methods section (further details in Supplementary Materials). The bioreactor produced in C8 composite material and externally coated in PDMS presented no water leaks in the watertight test after 24 h at 37°C, external or internal (infill space). The 24 h continuous perfusion test was successful, *i.e.*, the developed perfusion system sustained the required liquid level for that period. The individual operation of each applied sensor was confirmed in terms of stability and performance. Micro-computed tomography applied to the produced PCL orthogonal scaffold samples revealed a mean pore size of 490 ± 30 µm and a mean filament diameter of 518 ± 30 μm, which are within the values originally established for both properties.

**FIGURE 6:**
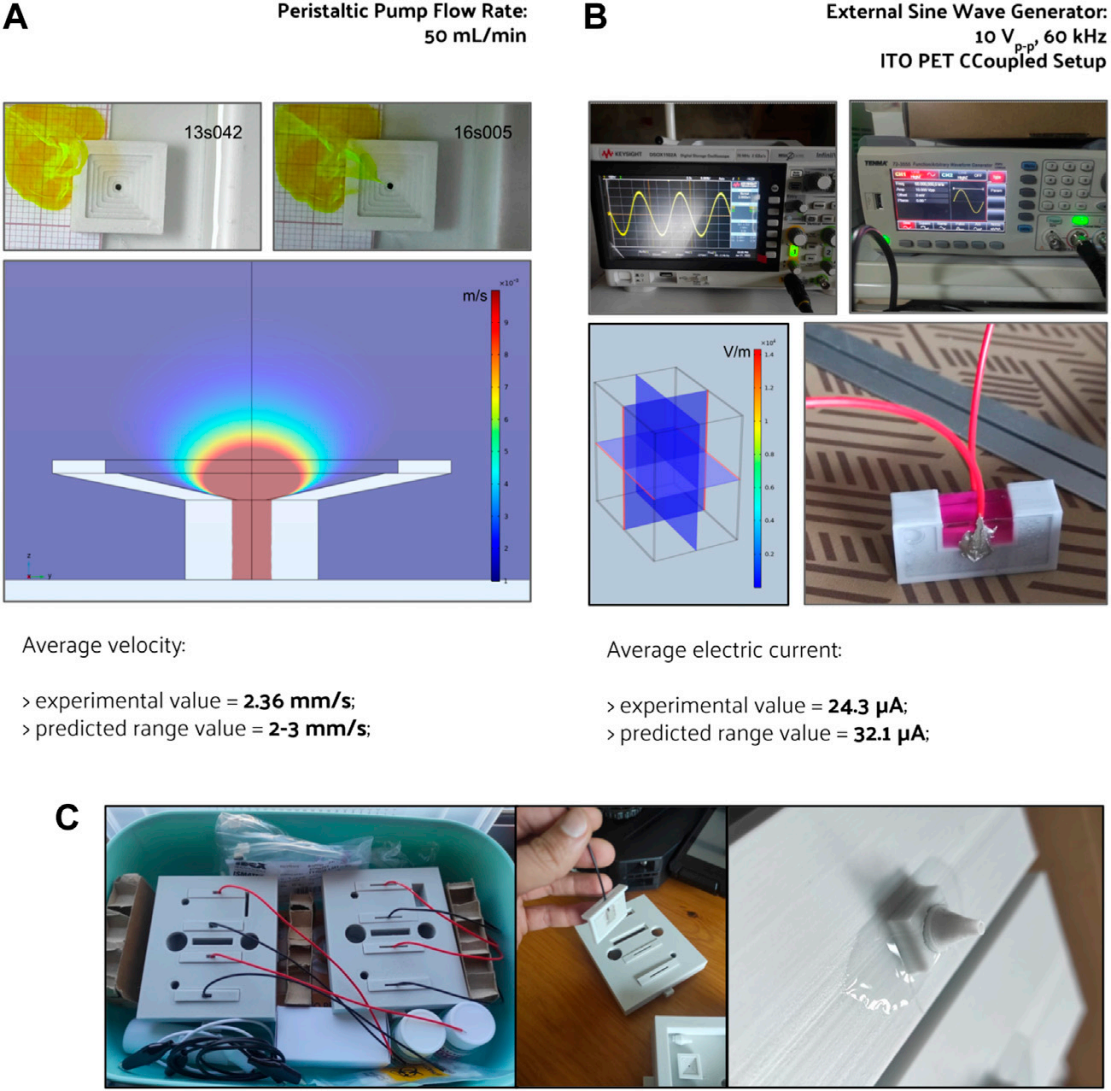
Pre-culture validation of the fabricated perfusion bioreactor, its CCoupled system, and their outputs comparison with the numerical model’s predictions for **(A)** fluid flow velocity and **(B)** electric current generated. The electric field generated by the CCoupled system validation setup was predicted to be uniform, with a value of 0.095 V m^−1^ at the culture medium region. **(C)** Photos from the assembled bioreactor, electrodes, and perfusion connectors.

Perfusion velocity measurements corresponded well to the numerical model predictions for the fluid flow at the bioreactor culture chamber without scaffold structures, as seen in Figure 6A. The two-time points, 13 s 042 ms, 16 s 005 ms, correspond to a yellow dye movement of 7 mm on top of the scaffold holder, which results in a mean velocity of 2.36 mm s^−1^, following the numerical prediction (2–3 mm s^−1^) for the same region and conditions.

Regarding the CCoupled stimulation system, the numerical model predicts an electric field of 0.095 V m^−1^ and an electric current of 3.21 × 10^−5^ A at the testing setup (using the material properties and values from Table 1). The experimental value of the electric current on the testing setup was 2.43 × 10^−5^ A (measured across a resistor with 21.89 k℧, Figure 6B). Using the developed FEM model and setting a floating potential boundary condition for these two currents, the model predicts an electric field magnitude of 0.072 V m^−1^ (for 24 µA) and 0.095 V m^−1^ (for 32 µA). A numerical prediction with a 24% difference for the same region and conditions, that at this scale could have been caused by model imprecisions in material properties. The developed CCoupled stimulation system model neglected the 21.89 k℧ resistor used to perform the measurement currents. If accounted for (by means of a circuit terminal boundary condition), it would drop the predicted electric field magnitude to 0.094 V m^−1^ (with a predicted current of 31.8 µA). Despite being neglected in the developed system model, due to its narrow impact, adaptations of this CCoupled stimulation system that change any of its main components should reconsider the measurement resistor impact.

### 3.3 *In vitro* cell culture validation

**FIGURE 7:**
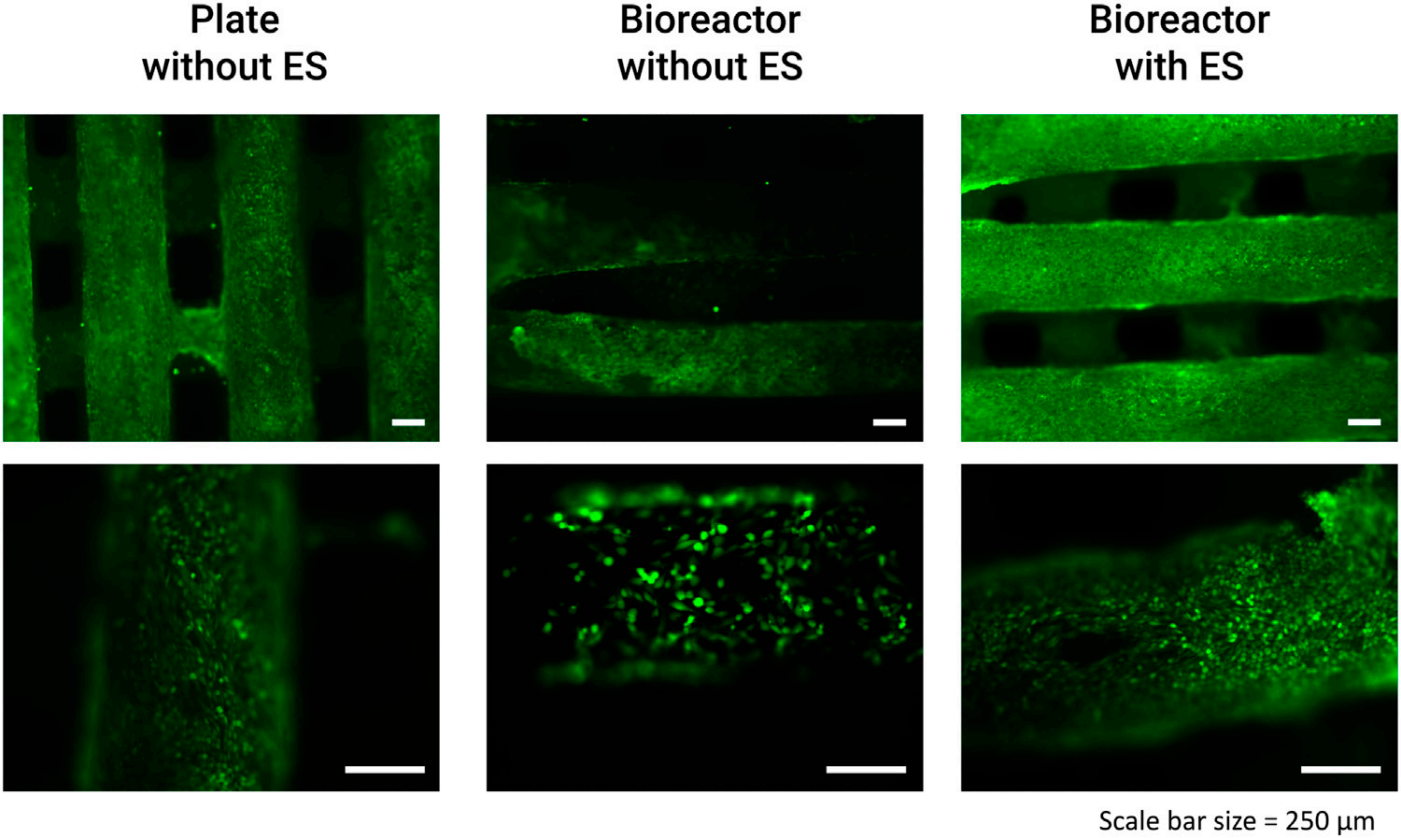
Results from the bioreactor preliminary human MG-63 osteoblasts cell culture viability tests performed with LIVE/DEAD staining at day 14 (after 3x CCoupled stimulation and 48 h bioreactor culture).

**FIGURE 8:**
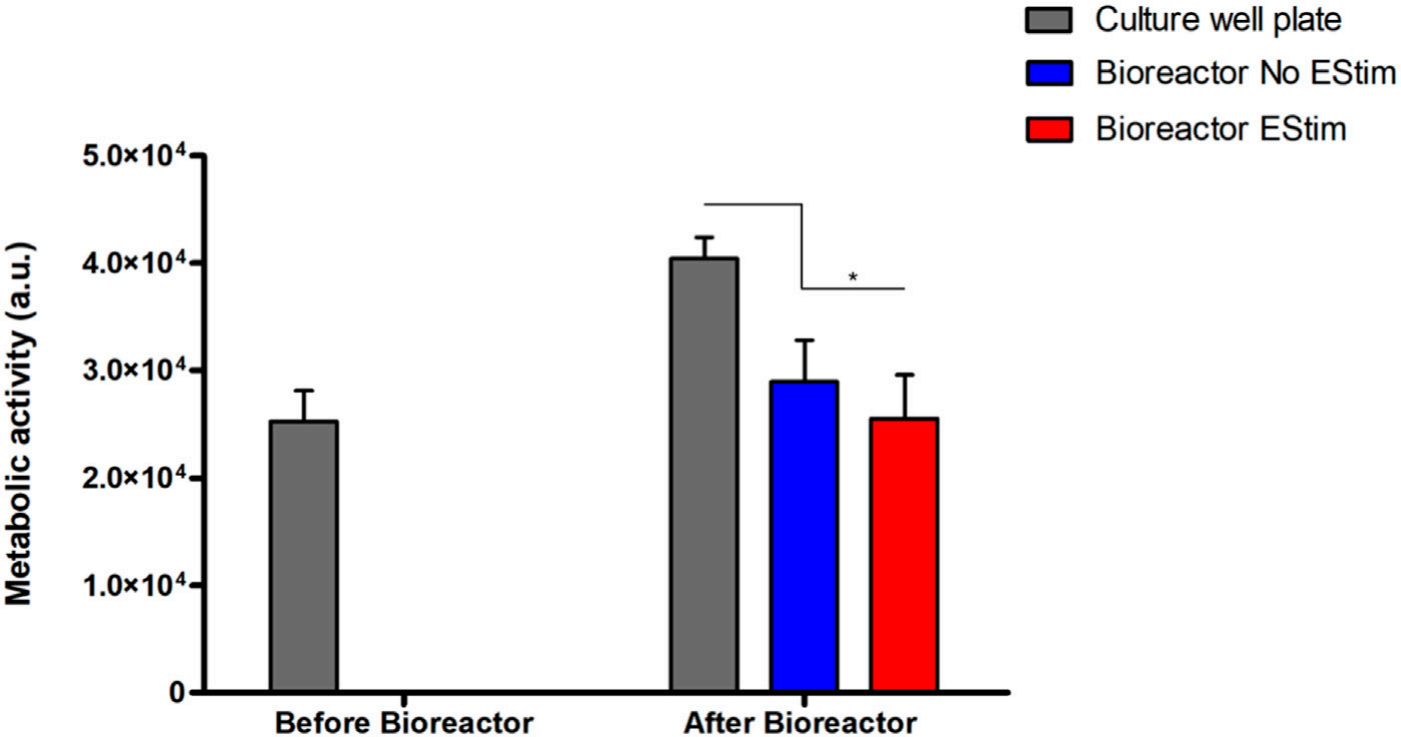
Results from the metabolic activity of human MG-63 osteoblasts on a bioreactor preliminary static cell culture. Results present time points before (Day 12) and after bioreactor culture (Day 14) with/without CCoupled stimulation. * account for significant differences *p* < 0.05.

The preliminary validation results of the fabricated perfusion bioreactor and its CCoupled system showed high cell viability and suggested no evidence of cell death in all conditions, as shown in Figure 7. Nonetheless, a significant effect is observed for this cell line when comparing cell-seeded scaffolds cultured in 24-well plates *versus* the bioreactor (no perfusion condition) cultures, with the latter presenting inferior metabolic activity (Figure 8) but maintaining high cell viability and spreading around the scaffold (Figure 7). The applied EF magnitude volume average of 0.118 V m^−1^ did not impact the viability of human MG-63 osteoblasts. Overall, the validation tests confirmed that the fabricated bioreactor design can support cell cultures without signs of cytotoxicity or reduced cellular viability.

## 4 Discussion

From a bird’s eye view, the developed bioreactor design is one of many possibilities that can be obtained if considering different starting points, actuation strategies, and goals to be achieved. Nonetheless, the development strategy ensures that the produced design complies with the application of predetermined stimulation environmental conditions, even when applied simultaneously, like simultaneous fluid-induced shear stress and electric field stimulation.

### 4.1 Decisions on bioreactor fabrication

We used numerical models to update the geometry or the stimulation protocol until established microenvironment properties were achieved. This iterative strategy is only possible if the resultant bioreactor design can be fabricated with high precision, a premise obtained through additive manufacturing technologies (Gensler et al., 2020). Various concepts of perfusion bioreactors (Birru et al., 2018; Smith et al., 2018; Daneshgar et al., 2019; Gabetti et al., 2022) have been 3D printed with stereolithography technology using class I biomaterial dental resin, while others have used fuse deposition modeling (FDM) technology (Raveling et al., 2018; Rosser and Thomas, 2018; Schmid et al., 2018). A challenge with FDM 3D prints is to obtain leakproof components since this process usually generates highly porous structures between filaments deposition. Different solutions were applied to overcome this issue. One approach was to cast an acrylonitrile butadiene styrene (ABS) part with a type of silicone rubber that cures at room temperature (commercially known as RTV silicone), creating a part mold and filling it with a two-part polyurethane casting resin (Rosser and Thomas, 2018; Schmid et al., 2018). Another approach was to 3D print the bioreactor in ABS and waterproof it by treating its parts with an acetone vapor bath (Raveling et al., 2018). In the present study, the bioreactor device was 3D printed, and afterward, all its 3D-printed pieces were coated with an external PDMS coating to achieve a watertight structure. Theoretically, it is possible to produce FDM leakproof parts without any added coating using larger nozzles and higher filament superposition. However, in a natural setting, this remains challenging due to small manufacturer imprecisions that occur during the printing process. Using an external coating allowed us to correct imprecisions that may have occurred during the 3D printing process.

### 4.2 Bioreactor inner environment

Some bioreactor-based strategies targetting bone regeneration are scaffoldless, like the one from Smith et al. (2018) that uses a Kenzan micro-needle array; others support a single scaffold or single tissue sample (Birru et al., 2018; Rosser and Thomas, 2018; Schmid et al., 2018; Visone et al., 2018; Daneshgar et al., 2019; Gabetti et al., 2022). Our design can support four scaffold structures under identical conditions in the same bioreactor. The operation concept behind our design is also flexible to expand to support even more scaffold structures.

The capability to monitor the fluid flow or electric phenomena in the presence of the scaffold structure and cellular content may be crucial to update and take more advantage of numerical models, extending their application into the monitoring and controlling of the cell culture microenvironment during the entire culture phase. Smith et al. (2018) integrated a Doppler ultrasound imaging system to assess the flow characteristics inside the bioreactor culture chamber. Abasi et al. (2020) proposed an electrical stimulation system that includes real-time monitoring of the response of a cellular membrane via AC electrical impedance spectroscopy. This design hypothesis may be fruitful in understanding electric changes in the cultured tissue. Our developed bioreactor uses commercially available sensors to perform real-time monitoring of pH, dissolved oxygen, and temperature fluctuations. However, there is still room for improvement regarding more accurate and less invasive procedures and/or miniaturized sensors.

Regarding multimodal stimulation approaches (fluid flow-induced shear stress and electromagnetic), the bioreactor designs from Gabetti et al. (2022) and Visone et al. (2018) are only two of the few examples of uni/bi-directional perfusion and simultaneous stimulation with a pulsed electromagnetic field (PEMF). Visone et al. (2018) also introduced an original design that allows a microscopic observation of the cell culture without exposing it to an external environment. Our proposed bioreactor approach similarly allows the delivery of uni/bi-directional perfusion, alone or combined with an electric field stimulation system, conceived as capacitively coupled to induce a pure electric field to the cell cultures inside the bioreactor and avoid cell toxicity due to faradaic products that can occur in direct-coupled systems. Our current design capabilities can be further expanded with actuators to control temperature and O_2_/CO_2_ gas concentrations, incorporating a system similar to the one described by Samokhin et al. (2022).

### 4.3 Bioreactor validation

Once fabricated and before the introduction of *in vitro* cell culture tests, the bioreactor was subjected to a pre-validation experiment to confirm if the conditions predicted in the numerical models are verified in the physical bioreactor system. Pre-culture validation results were in close correspondence with the experimental measures. Fluid flow velocity measurement was based on a video recording of a yellow dye wavefront movement, and the velocity was estimated using correspondent elapsed time between two known positions. This measurement was performed without a scaffold to improve the camera’s field of view over the dye flow. The recorded fluid flow velocity (2.36 mm s^−1^) matched the numerical predicted range for the same scaffoldless conditions at that same region (2–3 mm/s). Despite this agreement, more accurate techniques, like micro-particle image velocimetry (Guastamacchia et al., 2022) or ultrasound (Smith et al., 2018), could be applied to reconstruct velocity and shear stress fields in the presence of scaffold structures, allowing for comparison of CFD numerical models and experimental results with greater detail. Bone *in vivo* models predict that when under a typical loading of vigorous physiological activity, bone fluid flows with an approximate velocity of 6.50 × 10^−6^ m s^−1^ Verbruggen et al. (2014), a value obtainable with this bioreactor by dropping the peristaltic pump velocity about 100 times. Electric fields are difficult to measure directly, so we choose to determine their value by measuring the total electric current across a resistor in series with the CCoupled testing setup. A specially designed validation setup was built for the CCoupled system to spend less volume of culture medium while keeping identical conditions to the cell culture chamber without any scaffold or holder structures. The developed numerical model of this testing setup predicted a current of 3.21 × 10^−5^ A, higher than the measured value (2.43 × 10^−5^ A), but of the same order of magnitude. This slight overestimation may be caused by the material electrical properties adopted based on previous literature-reported values. This model should be further refined with electronic impedance spectrum characterization of the involved materials since impedance may relate to the applied signal frequency (Sanchez-Perez et al., 2022). Natural bone streaming potentials are thought to be in the proximity of 0.39 ± 0.14 mV Qin et al. (2002), electric potentials that can be currently generated in the ROI using the developed CCoupled system by lowering the amplitude of the applied sinusoidal wave.

Pre-culture validation was essential to confirm that numerical model predictions and experimental measures obtained were in close agreement. This approach serves two primary purposes: first, differences can help improve both models and design/fabrication procedures; second, matching values increases the confidence in our pipeline for bioreactor design and fabrication. Thus, we recommend that future studies using numerical models to fine-tune the 3D design of bioreactors should include pre-validation assessments.

### 4.4 Numerical modeling approaches

Multiple studies on bioreactor concepts introduced numerical models to predict the applied microenvironment conditions. Usually, these studies applied CFD models to empty chamber bioreactor digital designs to calculate flow regimes, velocity profile, and flow-induced shear stress (Rosser and Thomas, 2018; Schmid et al., 2018; Smith et al., 2018; Visone et al., 2018; Daneshgar et al., 2019; Lim et al., 2019; Gabetti et al., 2022). Gabetti et al. (2022) also performed electromagnetic field simulations to predict the distribution of the delivered magnetic field, and Visone et al. (2018) performed an electric field estimation very similar to the one performed in our work. Compared with our modeling strategy, these previous studies did not modify the bioreactor design interactively to adjust microenvironment predictions, thus limiting microenvironment adjustments using only external manipulation. Also, these works did not include models with scaffold structures, which are widely used in TE strategies and can powerfully shape fluid flow and electric field delivery. One improvement that may need to be addressed by future models is the need to introduce scaffold surface topology, since different scaffold surface properties will introduce local fluid flow-induced shear stresses that may be relevant in the generated biological response. Numerical models have commonly been used in isolation as alternatives to experimental measurements. However, with our approach, we demonstrate that it is a good practice to establish a VVUQ (verification–validation–uncertainty quantification) procedure when dealing with numerical models to cross-validate the model and bioreactor/scaffold fabrication before the cell culture studies phase.

### 4.5 *In vitro* cell culture tests

Preliminary cell culture validation was performed with human osteoblast-like MG-63 cells, a cell type usually applied in osteogenic-related studies Ghanbari et al. (2023); Dehkordi et al. (2022). Although cell viability remained impaired, cell metabolic activity was affected when the scaffolds with seeded cells were transferred from the well plate to the bioreactor. The decrease in metabolic activity was further enhanced by the application of a low-intensity electric field (0.118 V m^−1^), an effect also observed by Cohly et al. (2003) after applying a static electromagnetic field on the human MG-63 osteoblast cell line. MG-63 cells exposed to the static electromagnetic field were reported (Cohly et al., 2003) to have a 34% decrease in proliferation, 37% decrease in the secretion of proline, a significant component of collagen, and downregulation of collagen I, alkaline phosphatase, parathyroid hormone-receptor, and osteocalcin mRNAs. Cohly et al. (2003) concluded that exposure to very low static electric fields affects the human MG-63 osteoblasts in a manner that may be detrimental to bone formation. Our preliminary cell culture validation was performed under non-perfused conditions since our focus was first to test the cellular viability response when subjected to the EF from the developed CCoupled system compared to the well plate control conditions. CCoupled systems are used less often in TE applications than ubiquitous perfusion systems, justifying more attention in an initial application. Future work will include cell culture validations under different fluid flow profiles in combination with different electric field magnitudes to ascertain the definite effects on cell viability, proliferation, and osteogenic differentiation.

### 4.6 Reproducibility: Open-source solutions

Despite the diversity of bioreactor solutions currently available, to the best of our knowledge, only a handful of designs (Raveling et al., 2018; Daneshgar et al., 2019) have been shared open-source with complete fabrication blueprints. Most of the bioreactor designs found only briefly describe the fabrication methodology, usually with insufficient detail to allow its precise replication. This hampers reproducibility for other studies due to hard-to-guess geometrical dimensions and material properties, profoundly impacting microenvironmental conditions during external stimulation. As the extensive review from Nicksic et al. (2022) points out, retrieving conclusions from electric stimulation studies are challenging due to a substantial variability in protocol definition, stimulation conditions, and device specification reports, which are usually incomplete. We aim to overcome these issues by releasing all outputs from our JANUS approach under a public open-source Attribution-ShareAlike 4.0 International license (https://doi.org/10.5281/zenodo.7695700). We have included blueprints for the fabrication of this bioreactor/scaffold, electronic schematics, a detailed list of used components with their correspondent part reference, and all developed source files for numerical models (also shared with a complete report of its parametrization), control firmware and software. An overall cost estimate for fabricating a replica of this developed bioreactor is also presented in the Supplementary Table S3, along with a detailed development roadmap.

### 4.7 The main relevance of JANUS

JANUS combines a particular 3D printable bioreactor/scaffold design and its numerical models. This work focused on using this model prediction data-driven design strategy to recreate targeted cellular microenvironments. Introducing the scaffold structure into the bioreactor numerical model is critical to further understanding the conditions that have been created. Their geometry and material properties will become part of the cell microenvironment, affecting the way the fluid flows, thus shaping the mechanical stimulation (Zhao et al., 2018; Zhao et al., 2020; Moradkhani et al., 2021; Capuana et al., 2023) and affecting the way that electric/ionic currents might interfere with electrical stimulation delivery (Meneses et al., 2021). The generated microenvironment is expected to be as predicted by numerical models before cell seeding occurs. However, once cells are added to the scaffold, the microenvironment will change considerably due to cell proliferation and extracellular matrix secretion that fills the volumes once occupied by the culture medium, modifying scaffold topology and closing its pores. Previous studies have reported these effects and offer a window into the impact of cellular activity and growth on stimulation delivery (Zhao et al., 2020; Perier-Metz et al., 2021). These studies introduce cellular models into a chain of other existent models of bioreactor/scaffold microenvironment conditions, like the one we presented here, so stimulation delivery could be better predicted and modified, accounting for posterior cellular activity changes. Future work may elaborate on adding cellular models to this work baseline models and improve bioreactor design with control and actuation features, evenly introducing machine learning capabilities for real-time culture monitorization. Future work will also include long-term cell cultures to verify the performance of the developed bioreactor design for longer periods of time (from weeks to months).

The JANUS duality (virtual-physical) can still be helpful after the experimental setup design and stimulation protocol are set by helping to control and adjust the microenvironment to maintain the adequate *in vitro* conditions: for example, keeping an appropriate level of fluid-induced shear stress that induces differentiation of progenitor cells towards an osteoblastic lineage on 3D scaffolds in the absence of chemical stimulation (Yamada et al., 2021). Another advantage of the virtual-physical combination is to improve the comparison between protocols from different studies by allowing them to match other reported experimental conditions or serve as the baseline to feed further numerical models that account for cell seeding and neotissue metabolism, growth and proliferation (Reina-Romo et al., 2019).

## 5 Conclusion

In a nutshell, we presented a strategy to design multimodal bioreactors based on numerical model-driven decisions regarding the predicted microenvironment generated by the set of a particular bioreactor/scaffold geometries when applied with a specific stimulation protocol. Furthermore, we showed that combining physical and virtual approaches may improve the precision when constructing stimulation setups and applying cell culture stimulations, allowing for better control and overall replicability of experimental outcomes. The resulting perfusion bioreactor design was experimentally validated, capable of simultaneous mechanic and electric field stimulation. It is made available open source in an online platform with all its blueprints and fabrication instructions. This bioreactor virtual-physical strategy could also be helpful to drop experimental costs, allowing to optimize culture medium usage and the number of required bioreactors.

## Data Availability

The datasets presented in this study can be found in online repositories. The names of the repository/repositories and accession number(s) can be found in the article/Supplementary Material.

## References

[B1] Abasi S. Aggas J. R. Venkatesh N. Vallavanatt I. G. Guiseppi-Elie A. (2020). Design, fabrication and testing of an electrical cell stimulation and recording apparatus (ECSARA) for cells in electroculture. Biosens. Bioelectron. 147, 111793. 10.1016/j.bios.2019.111793 31669804

[B2] Alvarez-Barreto J. F. Landy B. VanGordon S. Place L. DeAngelis P. L. Sikavitsas V. I. (2011). Enhanced osteoblastic differentiation of mesenchymal stem cells seeded in RGD-functionalized PLLA scaffolds and cultured in a flow perfusion bioreactor. J. Tissue Eng. Regen. Med. 5, 464–475. 10.1002/term.338 20878644

[B3] Beşkardeş I. G. Aydın G. Bektaş Ş. Cengiz A. Gümüşderelioğlu M. (2018). A systematic study for optimal cell seeding and culture conditions in a perfusion mode bone-tissue bioreactor. Biochem. Eng. J. 132, 100–111. 10.1016/j.bej.2018.01.006

[B4] Bianconi S. Oliveira K. M. C. Klein K.-L. Wolf J. Schaible A. Schröder K. (2023). Pretreatment of mesenchymal stem cells with electrical stimulation as a strategy to improve bone tissue engineering outcomes. Cells 12, 2151. 10.3390/cells12172151 37681884 PMC10487010

[B5] Birru B. Mekala N. K. Parcha S. R. (2018). Improved osteogenic differentiation of umbilical cord blood MSCs using custom made perfusion bioreactor. Biomed. J. 41, 290–297. 10.1016/j.bj.2018.07.002 30580792 PMC6306301

[B6] Brighton C. T. Okereke E. Pollack S. R. Clark C. C. (1992). *In vitro* bone-cell response to a capacitively coupled electrical field. the role of field strength, pulse pattern, and duty cycle. Clin. Orthop. Relat. Res. 285, 255–262. 10.1097/00003086-199212000-00035 1446447

[B7] Budde K. Zimmermann J. Neuhaus E. Schröder M. Uhrmacher A. M. van Rienen U. (2019). “Requirements for documenting electrical cell stimulation experiments for replicability and numerical modeling,” in 2019 41st annual international conference of the IEEE engineering in medicine and biology society (EMBC), 1082–1088. 10.1109/EMBC.2019.8856863 31946082

[B8] Capuana E. Campora S. Catanzaro G. Lopresti F. Conoscenti G. Ghersi G. (2023). Computational modeling and experimental characterization of fluid dynamics in micro-CT scanned scaffolds within a multiple-sample airlift perfusion bioreactor. Biochem. Eng. J. 191, 108797. 10.1016/j.bej.2022.108797

[B9] Chen L. Deng C. Li J. Yao Q. Chang J. Wang L. (2019). 3D printing of a lithium-calcium-silicate crystal bioscaffold with dual bioactivities for osteochondral interface reconstruction. Biomaterials 196, 138–150. 10.1016/j.biomaterials.2018.04.005 29643002

[B10] Cohly H. H. P. Abraham G. E. 3rd Ndebele K. Jenkins J. J. Thompson J. Angel M. F. (2003). Effects of static electromagnetic fields on characteristics of MG-63 osteoblasts grown in culture. Biomed. Sci. Instrum. 39, 454–459. 12724935

[B11] Daneshgar A. Tang P. Remde C. Lommel M. Moosburner S. Kertzscher U. (2019). Teburu-Open source 3D printable bioreactor for tissue slices as dynamic three-dimensional cell culture models. Artif. Organs 43, 1035–1041. 10.1111/aor.13518 31211867

[B12] Dehkordi A. N. Shafiei S. S. Chehelgerdi M. Sabouni F. Sharifi E. Makvandi P. (2022). Highly effective electrospun polycaprolactone/layered double hydroxide nanofibrous scaffold for bone tissue engineering. J. Drug Deliv. Sci. Technol. 76, 103827. 10.1016/j.jddst.2022.103827

[B13] de Peppo G. M. Marcos-Campos I. Kahler D. J. Alsalman D. Shang L. Vunjak-Novakovic G. (2013). Engineering bone tissue substitutes from human induced pluripotent stem cells. Proc. Natl. Acad. Sci. U. S. A. 110, 8680–8685. 10.1073/pnas.1301190110 23653480 PMC3666730

[B14] Engel N. Fechner C. Voges A. Ott R. Stenzel J. Siewert S. (2021). An optimized 3d-printed perfusion bioreactor for homogeneous cell seeding in bone substitute scaffolds for future chairside applications. Sci. Rep. 11, 22228. 10.1038/s41598-021-01516-8 34782672 PMC8593024

[B15] Fernandes S. R. Meneses J. Datta A. Amado S. Alves N. Pascoal-Faria P. (2022). Comparison of electromagnetic stimulation fields generated by different experimental setups: a biophysical analysis. AIP Conf. Proc. 2425, 220004. 10.1063/5.0081338

[B16] Fitzsimmons R. J. Farley J. Adey W. R. Baylink D. J. (1986). Embryonic bone matrix formation is increased after exposure to a low-amplitude capacitively coupled electric field, *in vitro* . Biochim. Biophys. Acta 882, 51–56. 10.1016/0304-4165(86)90054-1 3707998

[B17] Gabetti S. Masante B. Cochis A. Putame G. Sanginario A. Armando I. (2022). An automated 3d-printed perfusion bioreactor combinable with pulsed electromagnetic field stimulators for bone tissue investigations. Sci. Rep. 12, 13859. 10.1038/s41598-022-18075-1 35974079 PMC9381575

[B18] Gaspar D. A. Gomide V. Monteiro F. J. (2012). The role of perfusion bioreactors in bone tissue engineering. Biomatter 2, 167–175. 10.4161/biom.22170 23507883 PMC3568103

[B19] Gensler M. Leikeim A. Möllmann M. Komma M. Heid S. Müller C. (2020). 3D printing of bioreactors in tissue engineering: a generalised approach. PLoS One 15, e0242615. 10.1371/journal.pone.0242615 33253240 PMC7703892

[B20] Geris L. Lambrechts T. Carlier A. Papantoniou I. (2018). The future is digital: *in silico* tissue engineering. Curr. Opin. Biomed. Eng. 6, 92–98. 10.1016/j.cobme.2018.04.001

[B21] Ghanbari E. Khazaei M. Mehdipour A. Khoshfeterat A. Niknafs B. (2023). Green synthesized magnesium oxide nanoparticles reinforce osteogenesis properties of bacterial cellulose scaffolds for bone tissue engineering applications: an *in vitro* assessment. Cell J. 25, 483–495. 10.22074/cellj.2023.1986179.1204 37543861 PMC10404355

[B22] Guastamacchia M. G. R. Xue R. Madi K. Pitkeathly W. T. E. Lee P. D. Webb S. E. D. (2022). Instantaneous 4D micro-particle image velocimetry (µPIV) via multifocal microscopy (MUM). Sci. Rep. 12, 18458. 10.1038/s41598-022-22701-3 36323775 PMC9630545

[B23] Haleem A. Javaid M. Khan R. H. Suman R. (2020). 3D printing applications in bone tissue engineering. J. Clin. Orthop. Trauma 11, S118–S124. 10.1016/j.jcot.2019.12.002 31992931 PMC6977158

[B24] Hayashi K. Kishida R. Tsuchiya A. Ishikawa K. (2019). Carbonate apatite micro-honeycombed blocks generate bone marrow-like tissues as well as bone. Adv. Biosyst. 3, e1900140. 10.1002/adbi.201900140 32648680

[B25] Hayashi K. Munar M. L. Ishikawa K. (2020). Effects of macropore size in carbonate apatite honeycomb scaffolds on bone regeneration. Mat. Sci. Eng. C Mat. Biol. Appl. 111, 110848. 10.1016/j.msec.2020.110848 32279778

[B26] Hayashi K. Shimabukuro M. Kishida R. Tsuchiya A. Ishikawa K. (2022). Structurally optimized honeycomb scaffolds with outstanding ability for vertical bone augmentation. J. Adv. Res. 41, 101–112. 10.1016/j.jare.2021.12.010 36328740 PMC9637481

[B27] Hegde V. J. Gallot-Lavallee O. Heux L. (2015). Overview on thermal and electrical properties of biodegradable polymers.

[B28] Hidalgo-Bastida L. A. Thirunavukkarasu S. Griffiths S. Cartmell S. H. Naire S. (2012). Modeling and design of optimal flow perfusion bioreactors for tissue engineering applications. Biotechnol. Bioeng. 109, 1095–1099. 10.1002/bit.24368 22068720

[B29] Klein S. G. Alsolami S. M. Steckbauer A. Arossa S. Parry A. J. Ramos Mandujano G. (2021). A prevalent neglect of environmental control in mammalian cell culture calls for best practices. Nat. Biomed. Eng. 5, 787–792. 10.1038/s41551-021-00775-0 34389822

[B30] Klein S. G. Steckbauer A. Alsolami S. M. Arossa S. Parry A. J. Li M. (2022). Toward best practices for controlling mammalian cell culture environments. Front. Cell Dev. Biol. 10, 788808. 10.3389/fcell.2022.788808 35265608 PMC8900666

[B31] Korenstein R. Somjen D. Fischler H. Binderman I. (1984). Capacitative pulsed electric stimulation of bone cells. induction of cyclic-AMP changes and DNA synthesis. Biochim. Biophys. Acta 803, 302–307. 10.1016/0167-4889(84)90121-6 6322860

[B32] LaNasa P. J. Loy Upp E. (2014). Fluid flow measurement: a practical guide to accurate flow measurement. Butterworth-Heinemann.

[B33] Lim D. Renteria E. S. Sime D. S. Ju Y. M. Kim J. H. Criswell T. (2022). Bioreactor design and validation for manufacturing strategies in tissue engineering. Biodes Manuf. 5, 43–63. 10.1007/s42242-021-00154-3 35223131 PMC8870603

[B34] Lim K.-T. Patel D. K. Seonwoo H. Kim J. Chung J. H. (2019). A fully automated bioreactor system for precise control of stem cell proliferation and differentiation. Biochem. Eng. J. 150, 107258. 10.1016/j.bej.2019.107258

[B35] Marolt D. Campos I. M. Bhumiratana S. Koren A. Petridis P. Zhang G. (2012). Engineering bone tissue from human embryonic stem cells. Proc. Natl. Acad. Sci. U. S. A. 109, 8705–8709. 10.1073/pnas.1201830109 22586099 PMC3365157

[B36] Meneses J. C Silva J. R Fernandes S. Datta A. Castelo Ferreira F. Moura C. (2020). A multimodal stimulation cell culture bioreactor for tissue engineering: a numerical modelling approach. Polymers 12, 940. 10.3390/polym12040940 32325660 PMC7240379

[B37] Meneses J. Fernandes S. Alves N. Pascoal-Faria P. Miranda P. C. (2022a). Author correction: how to correctly estimate the electric field in capacitively coupled systems for tissue engineering: a comparative study. Sci. Rep. 12, 12522. 10.1038/s41598-022-16724-z 35869237 PMC9307844

[B38] Meneses J. Fernandes S. R. Alves N. Pascoal-Faria P. Miranda P. C. (2021). Effects of scaffold electrical properties on electric field delivery in bioreactors. Conf. Proc. IEEE Eng. Med. Biol. Soc. 2021, 4147–4151. 10.1109/EMBC46164.2021.9630711 34892139

[B39] Meneses J. Fernandes S. R. Datta A. Amado S. Alves N. Pascoal-Faria P. (2022b). Numerical modelling of a bioreator design targeting optimal conditions for cell culture. AIP Conf. Proc. 2425, 220003. 10.1063/5.0081336

[B40] MIT (2023). Review of material properties. n.d. 6.777j/2.751j material properties database.

[B41] Monfoulet L.-E. Becquart P. Marchat D. Vandamme K. Bourguignon M. Pacard E. (2014). The ph in the microenvironment of human mesenchymal stem cells is a critical factor for optimal osteogenesis in tissue-engineered constructs. Tissue Eng. Part A 20, 1827–1840. 10.1089/ten.tea.2013.0500 24447025

[B42] Montorsi M. Genchi G. G. De Pasquale D. De Simoni G. Sinibaldi E. Ciofani G. (2022). Design, fabrication, and characterization of a multimodal reconfigurable bioreactor for bone tissue engineering. Biotechnol. Bioeng. 119, 1965–1979. 10.1002/bit.28100 35383894 PMC9324218

[B43] Moradkhani M. Vahidi B. Ahmadian B. (2021). Finite element study of stem cells under fluid flow for mechanoregulation toward osteochondral cells. J. Mat. Sci. Mat. Med. 32, 84. 10.1007/s10856-021-06545-3 PMC8266696 34236534

[B44] Morgan E. F. Unnikrisnan G. U. Hussein A. I. (2018). Bone mechanical properties in healthy and diseased states. Annu. Rev. Biomed. Eng. 20, 119–143. 10.1146/annurev-bioeng-062117-121139 29865872 PMC6053074

[B45] Nicksic P. J. Donnelly D. T. Hesse M. Bedi S. Verma N. Seitz A. J. (2022). Electronic bone growth stimulators for augmentation of osteogenesis in *in vitro* and *in vivo* models: a narrative review of electrical stimulation mechanisms and device specifications. Front. Bioeng. Biotechnol. 10, 793945. 10.3389/fbioe.2022.793945 35237571 PMC8882968

[B46] Perier-Metz C. Duda G. N. Checa S. (2021). Initial mechanical conditions within an optimized bone scaffold do not ensure bone regeneration - an *in silico* analysis. Biomech. Model. Mechanobiol. 20, 1723–1731. 10.1007/s10237-021-01472-2 34097188 PMC8450217

[B47] Pollard D. A. Pollard T. D. Pollard K. S. (2019). Empowering statistical methods for cellular and molecular biologists. Mol. Biol. Cell 30, 1359–1368. 10.1091/mbc.e15-02-0076 31145670 PMC6724699

[B48] Qin Y.-X. Lin W. Rubin C. (2002). The pathway of bone fluid flow as defined by *in vivo* intramedullary pressure and streaming potential measurements. Ann. Biomed. Eng. 30, 693–702. 10.1114/1.1483863 12108843

[B49] Raveling A. R. Theodossiou S. K. Schiele N. R. (2018). A 3D printed mechanical bioreactor for investigating mechanobiology and soft tissue mechanics. MethodsX 5, 924–932. 10.1016/j.mex.2018.08.001 30167382 PMC6111048

[B50] Reina-Romo E. Papantoniou I. Bloemen V. Geris L. (2019). “4 - computational design of tissue engineering scaffolds,” in Handbook of tissue engineering scaffolds: volume one. Editors Mozafari M. Sefat F. Atala A. (Sawston, Cambridge: Woodhead Publishing), 73–92.

[B51] Rosser J. Thomas D. J. (2018). “10 - bioreactor processes for maturation of 3D bioprinted tissue,” in 3D bioprinting for reconstructive surgery. Editors Thomas D. J. Jessop Z. M. Whitaker I. S. (Sawston, Cambridge: Woodhead Publishing), 191–215.

[B52] Samokhin P. Gardner G. L. Moffatt C. Stuart J. A. (2022). An inexpensive incubator for mammalian cell culture capable of regulating o2, CO2, and temperature.

[B53] Sanchez-Perez A. Limones-Ahijon B. Garcia-Martin J. M. Sagardoy M. U. G. Mobini S. (2022). Electrochemical aspects of *in vitro* electrical stimulation devices.

[B54] Schmid J. Schwarz S. Meier-Staude R. Sudhop S. Clausen-Schaumann H. Schieker M. (2018). A perfusion bioreactor system for cell seeding and Oxygen-Controlled cultivation of Three-Dimensional cell cultures. Tissue Eng. Part C Methods 24, 585–595. 10.1089/ten.tec.2018.0204 30234443 PMC6208160

[B55] Schröder M. Reseland J. E. Haugen H. J. (2022). Osteoblasts in a perfusion flow bioreactor-tissue engineered constructs of TiO2 scaffolds and cells for improved clinical performance. Cells 11, 1995. 10.3390/cells11131995 35805079 PMC9265932

[B56] Seddiqi H. Saatchi A. Amoabediny G. Helder M. N. Ravasjani S. A. Aghaei M. S. H. (2020). Inlet flow rate of perfusion bioreactors affects fluid flow dynamics, but not oxygen concentration in 3d-printed scaffolds for bone tissue engineering: computational analysis and experimental validation. Comput. Biol. Med. 124, 103826. 10.1016/j.compbiomed.2020.103826 32798924

[B57] Shakeel M. Matthews P. C. Graham R. S. Waters S. L. (2013). A continuum model of cell proliferation and nutrient transport in a perfusion bioreactor. Math. Med. Biol. 30, 21–44. 10.1093/imammb/dqr022 21994793

[B58] Shibahara K. Hayashi K. Nakashima Y. Ishikawa K. (2022). Effects of channels and micropores in honeycomb scaffolds on the reconstruction of segmental bone defects. Front. Bioeng. Biotechnol. 10, 825831. 10.3389/fbioe.2022.825831 35372306 PMC8971796

[B59] Silva J. C. Carvalho M. S. Udangawa R. N. Moura C. S. Cabral J. M. S. L da Silva C. (2020). Extracellular matrix decorated polycaprolactone scaffolds for improved mesenchymal stem/stromal cell osteogenesis towards a patient-tailored bone tissue engineering approach. J. Biomed. Mat. Res. B Appl. Biomater. 108, 2153–2166. 10.1002/jbm.b.34554 31916699

[B60] Sladkova M. De Peppo G. M. (2014). Bioreactor systems for human bone tissue engineering. Processes 2, 494–525. 10.3390/pr2020494

[B61] Smith L. J. Li P. Holland M. R. Ekser B. (2018). FABRICA: a bioreactor platform for printing, perfusing, observing, and stimulating 3D tissues. Sci. Rep. 8, 7561. 10.1038/s41598-018-25663-7 29765087 PMC5953945

[B62] Spencer T. J. Hidalgo-Bastida L. A. Cartmell S. H. Halliday I. Care C. M. (2013). *In silico* multi-scale model of transport and dynamic seeding in a bone tissue engineering perfusion bioreactor. Biotechnol. Bioeng. 110, 1221–1230. 10.1002/bit.24777 23124479

[B63] Stephan M. Zimmermann J. Klinder A. Sahm F. van Rienen U. Kämmerer P. W. (2020). Establishment and evaluation of an *in vitro* system for biophysical stimulation of human osteoblasts. Cells 9, 1995. 10.3390/cells9091995 32872592 PMC7564340

[B64] Tandon N. Marsano A. Cannizzaro C. Voldman J. Vunjak-Novakovic G. (2008). Design of electrical stimulation bioreactors for cardiac tissue engineering. Conf. Proc. IEEE Eng. Med. Biol. Soc. 2008, 3594–3597. 10.1109/IEMBS.2008.4649983 PMC2771167 19163486

[B65] Touri M. Moztarzadeh F. Osman N. A. A. Dehghan M. M. Mozafari M. (2018). 3d-printed biphasic calcium phosphate scaffolds coated with an oxygen generating system for enhancing engineered tissue survival. Mat. Sci. Eng. C Mat. Biol. Appl. 84, 236–242. 10.1016/j.msec.2017.11.037 29519434

[B66] Verbruggen S. W. Vaughan T. J. McNamara L. M. (2014). Fluid flow in the osteocyte mechanical environment: a fluid-structure interaction approach. Biomech. Model. Mechanobiol. 13, 85–97. 10.1007/s10237-013-0487-y 23567965

[B67] Vetsch J. R. Betts D. C. Müller R. Hofmann S. (2017). Flow velocity-driven differentiation of human mesenchymal stromal cells in silk fibroin scaffolds: a combined experimental and computational approach. PLoS One 12, e0180781. 10.1371/journal.pone.0180781 28686698 PMC5501602

[B68] Visone R. Talò G. Lopa S. Rasponi M. Moretti M. (2018). Enhancing all-in-one bioreactors by combining interstitial perfusion, electrical stimulation, on-line monitoring and testing within a single chamber for cardiac constructs. Sci. Rep. 8, 16944. 10.1038/s41598-018-35019-w 30446711 PMC6240103

[B69] Wilson D. I. (2018). What is rheology? Eye 32, 179–183. 10.1038/eye.2017.267 29271417 PMC5811736

[B70] Wittkowske C. Reilly G. C. Lacroix D. Perrault C. M. (2016). *In vitro* bone cell models: impact of fluid shear stress on bone formation. 10.3389/fbioe.2016.00087 PMC5108781 27896266

[B71] Yamada S. Yassin M. A. Schwarz T. Hansmann J. Mustafa K. (2021). Induction of osteogenic differentiation of bone marrow stromal cells on 3D polyester-based scaffolds solely by subphysiological fluidic stimulation in a laminar flow bioreactor. 10.1177/20417314211019375 PMC8243246 34262684

[B72] Yamada S. Yassin M. A. Schwarz T. Mustafa K. Hansmann J. (2022). Optimization and validation of a Custom-Designed perfusion bioreactor for bone tissue engineering: flow assessment and optimal culture environmental conditions. Front. Bioeng. Biotechnol. 10, 811942. 10.3389/fbioe.2022.811942 35402393 PMC8990132

[B73] Zhao F. Lacroix D. Ito K. van Rietbergen B. Hofmann S. (2020). Changes in scaffold porosity during bone tissue engineering in perfusion bioreactors considerably affect cellular mechanical stimulation for mineralization. Bone Rep. 12, 100265. 10.1016/j.bonr.2020.100265 32613033 PMC7315008

[B74] Zhao F. van Rietbergen B. Ito K. Hofmann S. (2018). Flow rates in perfusion bioreactors to maximise mineralisation in bone tissue engineering *in vitro* . J. Biomech. 79, 232–237. 10.1016/j.jbiomech.2018.08.004 30149981

