# Supplementary material for: JANUS: an open-source 3D printable perfusion bioreactor and numerical model-based design strategy for tissue engineering
Source: Front Bioeng Biotechnol. 2023 Dec 15;11:1308096. doi: 10.3389/fbioe.2023.1308096 (PMC10757336; doi:10.3389/fbioe.2023.1308096)
Supplement: Supplementary file 1 [file DataSheet1.PDF]

Supplementary Materials.

An overview of the bioreactor development roadmap is available in Figure S1 and guides the overall structure of this supplementary materials document. A detailed description of each step of the roadmap follows the overview, a description that complements in greater detail the original description made in the main manuscript.

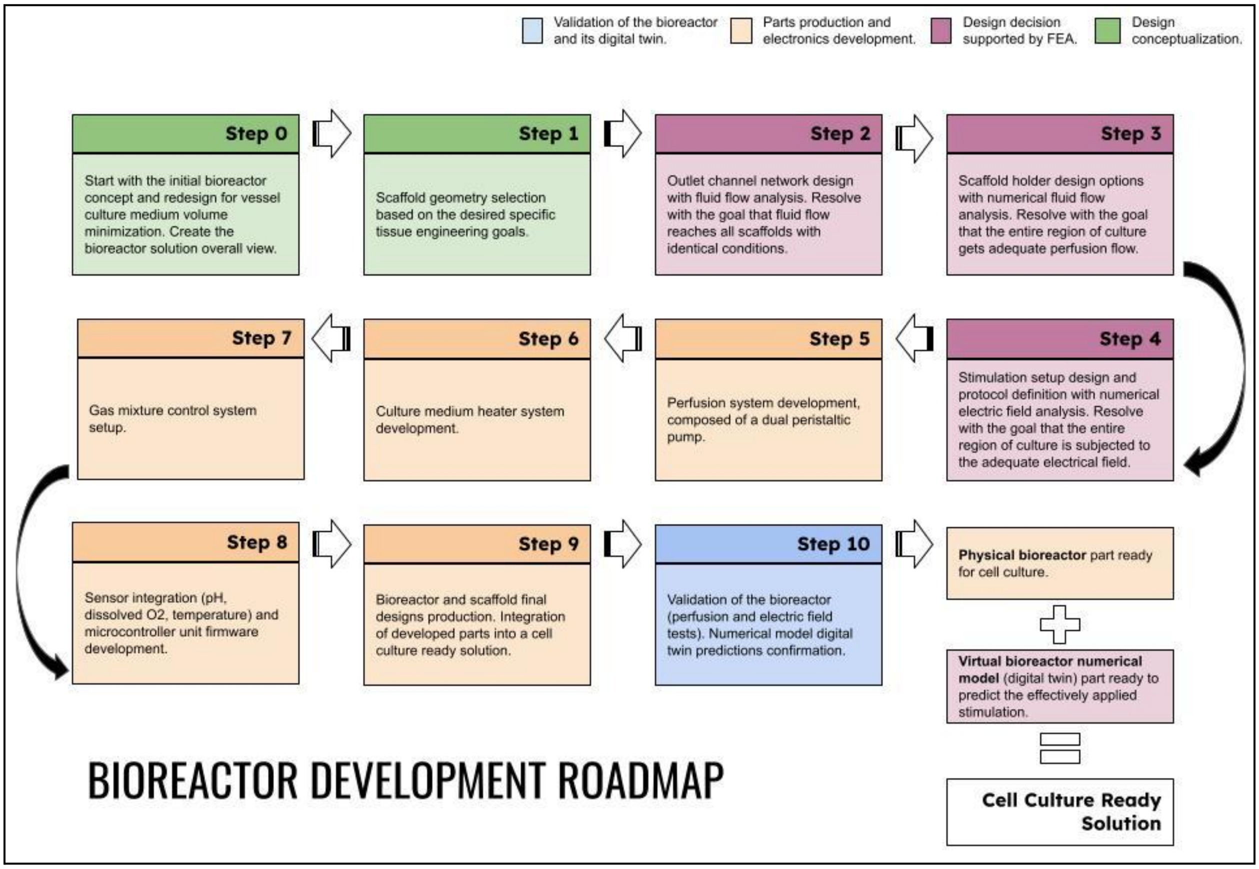

Figure S1 - Bioreactor development roadmap schematic with a step-by-step description alternating between design development and fabrication tasks, where numerical model predictions mainly support design decisions.

Vessel culture medium volume minimization **(Step 0)**.

The starting point for this development was a previously bioreactor concept progressively modified to integrate multiple stimulation modes without losing the ability to be entirely 3D printable. Table S1 lists the main features and evolution of the bioreactor design until its 9th version. This version was selected as the starting point for this work. The liquid volume for this starting point version was calculated with SOLIDWORKS and found to be 554 cm^3. The heater module present in previous versions was removed from the main chamber, freeing up space, minimizing the culture medium volume in the current bioreactor design update, and decreasing its utilization-associated cell culture costs.

Additionally, the height of the bioreactor was shortened, and a new central inlet channel was created. The bioreactor lid also includes inner bulk structures to reduce and guide fluid flow from the main inlet to the scaffold holders. The position of all sensors and electrodes was distributed at the developed lid, minimizing the required surrounding volume while guaranteeing proper readings.

Scaffold geometry selection **(Step 1)**.

Two scaffold geometries were selected as examples to apply the described development methodology. Our selection criteria focused on bone regeneration scaffold geometries that have been actively researched and can match some mechanical properties of cortical or trabecular bone formations. Numerical analyses were performed, including both selected scaffold geometries, after which the scaffold that predictably allowed a more osteogenic environment was selected for fabrication and further validation.

Table S1 - Development timeline of the bioreactor until the 9th version that was used as the starting point for this work bioreactor update.

	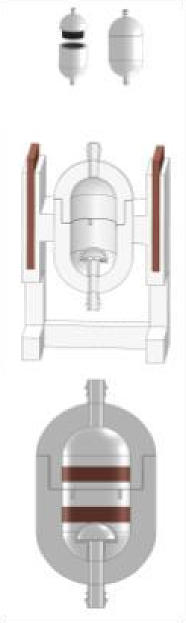	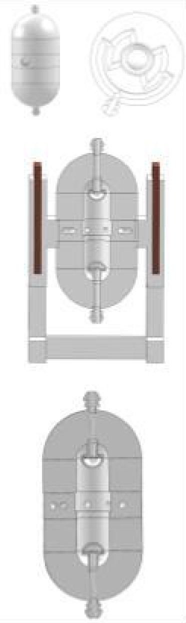	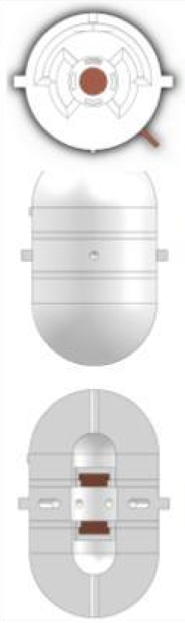	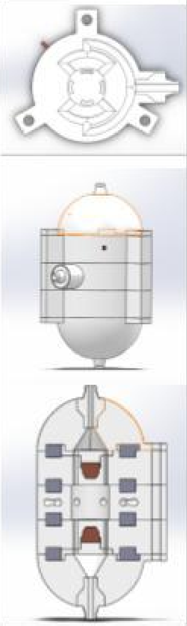	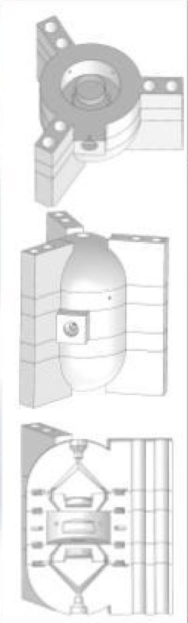	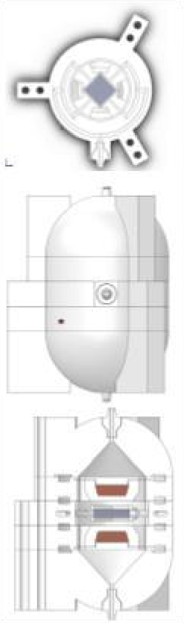	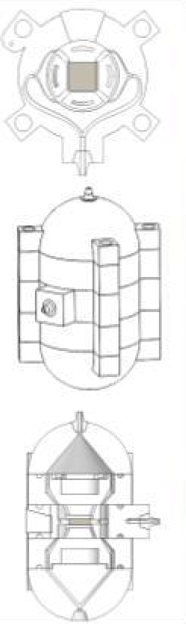	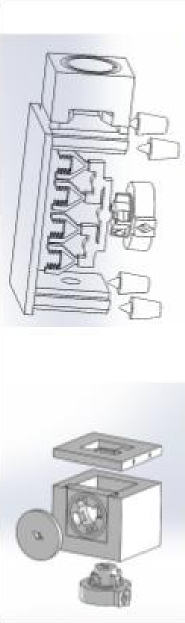	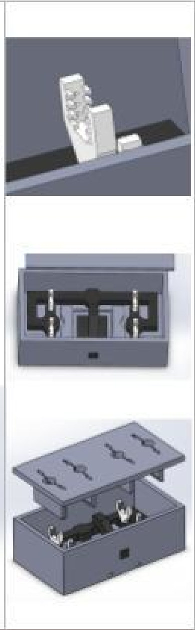
Version Number	1st	2nd	3rd	4th	5th	6th	7th	8th	9th
Main Feature(s)	>Single inlet and outlet;<br>> Flow splitter in a "Umbrella" shape;<br>> Supports external capacitively coupled electrodes (parallel plates) or internal direct coupled electrodes (rings);<br>>Single scaffold culture;	> Dual inlet and single outlet (radial system);<br>> Flow splitter in a "Umbrella" shape;<br>> Supports external capacitively coupled electrodes (parallel plates) or internal direct coupled electrodes (rings);<br>>Single scaffold culture;	> Dual inlet and single outlet (radial system);<br>> Flow splitter wall added to uniformize inlet fluid flow;<br>> Electrodes moved internally to avoid thick bioreactor structures;<br>> Supports CCoupled or DCoupled electrodes;<br>>Single scaffold culture;	> Adapted for FFF 3D printing production (modular design);<br>> M6 screws support for module fixation added;<br>> Hose joints improved;<br>> Dual inlet and single outlet (radial system);<br>> Supports CCoupled or DCoupled electrodes;<br>>Single scaffold culture;	> Inlet channel and flow splitter geometry improved for osteogenic flow;<br>> Adapted for FFF 3D printing production (modular design);<br>> Dual inlet and single outlet (radial system);<br>> Supports CCoupled or DCoupled electrodes;<br>>Single scaffold culture;	> Two halves assembly;<br>> Adapted for FFF 3D printing production (modular design);<br>> Dual inlet and single outlet (radial system);<br>> Supports CCoupled or DCoupled electrodes;<br>>Single scaffold culture;	> Radial outlet systems with a new design;<br>> Cell seeding door added;<br>> Adapted for FFF 3D printing production (modular design);<br>> Dual inlet and single outlet (radial system);<br>> Supports CCoupled or DCoupled electrodes;<br>>Single scaffold culture;	> Single inlet and single outlet (holder system);<br>>Four scaffold culture;<br>> Adapted for FFF 3D printing production (modular design);<br>> Supports CCoupled or DCoupled electrodes;<br>> Integrated heater;	> Single inlet and single outlet (holder system);<br>>Four scaffold culture;<br>> Adapted for FFF 3D printing production (modular design);<br>> Supports CCoupled or DCoupled electrodes;<br>> Integrated heater;
Major Update(s)	Initial concept.	Fluid flow symmetry at the culture region with the dual inlets and radial outlet system.	Capable of perfusion fluid flow shear stress and DCoupled electric stimulation in osteogenic ranges.	Translation from concept to 3D printing production. Modular interchangeable and adaptive design created.	Inlet channels optimization for osteogenic conditions regarding commercial peristaltic pumps flow.	Module improvement, design in two halves to allow for manual cell seeding and scaffold position with holders.	New manual cell seeding access throw a backdoor in the central module. Concept adaptation. New radial outlet channels.	Redesign to improve watertightness. Creating locker connectors to insert multiple inlet/outlet, sensors. Heating added.	Further redesign to improve watertightness. Less modules and module connections.
Development Stage	Concept, Resin Prototype. (Jun 2019)	Concept. (Oct 2019)	Concept. FEA. Meneses et al 2020.	Concept. Prototype in C8 material.(Apr 2020)	Concept. FEA Meneses et al (published in 2022)	Concept. Prototype in C8 material. (Dec 2020)	Concept. Prototype in C8 material. Laboratory tested. (Jun 2021)	Concepts. Prototype in C8 material. (Mar 2022)	Concept. Prototype in C8 material. (Sept 2022)

Outlet channel network design with numerical fluid flow analysis **(Step 2)**.

It was required to design and implement a proper channel network to support a predetermined number of scaffolds per bioreactor and provide each one with identical perfusion flow characteristics. We selected to support four scaffolds per bioreactor since usual testing conditions in TE consider several identical samples to be higher than three (N>3) to obtain reasonable statistical power. The developed channel network goal was to divide the outlet flow equally among all supported scaffolds, guaranteeing an equal pressure drop. The proposed design achieved that by following a simple rule: at every channel split, the section area of the child branches must be equal to the section area of the parent branch. A cyclic iteration between CAD design and CFD was conducted until one design average velocity magnitude estimated at the scaffold holder connector surface was higher than 50% of the imposed outlet velocity. At that point, the design phase was complete. Numerical predictions were performed considering the higher outlet velocity that the developed peristaltic pump device could provide, which was 50 ml/min (corresponding to 0.3281 m/s at the outlet) when mounted with a Tygon LMT55 3-Stop (Cole-Parmer GmbH, Germany) peristaltic tube with an internal diameter of 2.79 mm.

[Calculation Steps]:
conversions = 1 ml = 1 cm3; 1 min = 60 s; maximum_v = 50 ml/min = 50 cm3/min = 50/60 cm3/s; hose_area = pi(1.8/2)^2 = 2.54 mm2 = 0.0254 cm2;*
*hose_v = 50/(60*0.0254) = 32.81 cm/s = 0.3281 m/s (hose);*

Scaffold holder design options with numerical fluid flow analysis **(Step 3)**.

Two base design options for scaffold holders were conceived to support the selected scaffold geometries in different positions (vertical vs. horizontal). Both holder channel geometries were adapted to obtain high perfusion flows at the scaffold region, following the limitation given by the peristaltic pump maximum operation flow rate and also 3D printing design constraints. Combining two holder hypotheses with two selected scaffold options resulted in four different numerical models, where each selected scaffold was placed in its reserved holder position. For COMSOL analysis, a culture region was established with the scaffold's external envelope size. To represent the culture medium inside the developed bioreactor chamber, a cylindrical volume of a radius of 20 mm and height of 30 mm was added surrounding the holder and scaffold structures. As stated, a FEM model was constructed for each combination of holder and scaffold with inlet boundary conditions added to the surrounding cylindrical volume surfaces, defined as standard atmosphere pressure (101.325 Pa). A single outlet boundary condition was added to the holder exit channel circular surface (made with equal dimensions in both holder design options) with the average velocity magnitude value obtained from the numerical predictions of the previous step 2. The CFD mesh and model were then computed. Data post-processing was performed for each scaffold region, and plots with the volumetric distribution of Reynolds number, fluid-induced shear stress, fluid flow overall velocity, and axial component velocity magnitude were constructed and analyzed. Fluid-induced shear stress was calculated from COMSOL shear rate results and water dynamic viscosity at 37°C, an approximation valid for Newtonian fluids as derived from the following expression:

[Calculation Steps]: Wall Shear Stress(@37°C) = Dynamic Viscosity(@37°C)*Shear Rate;

CCoupled electrode development. Electric field stimulation protocol definition guided by numerical analysis **(Step 4)**.

Two capacitive coupled electrodes composed of ITO PET films an indium tin oxide coated 175-micrometre polyester film (60 ohm/sq) that was glued to a 3D printed structure with polydimethylsiloxane (Sylgard 184 Silicone Elastomer Kit, applied in a 10:1 (w/w) ratio of base to curing agent) and left to dry overnight. This structure was made with proprietary C8 material (3D4Makers, Netherlands) and printed with an Ender 3 S1 Pro 3D FFF printer (Creality, China). The printer specifications were set accordingly with the C8 manufacturer datasheet (printing temperature: 210°C, bed temperature: 50°C, maximum printing speed: 35 mm/s). A copper wire was connected to the conducting side of the ITO PET film with silver conductive epoxy adhesive (8331D, MG Chemicals). The constructed ITO PET capacitive coupled electrodes (33x18 mm) were modeled along with the developed holders and selected scaffolds, resulting in four distinct models. The electrodes were placed in parallel positions and equidistant from the scaffold center, with a 22 mm distance between them. The applied external electric potential was set to the maximum output of the available laboratory waveform generator (Tektronix, AFG2022 Arbitrary Waveform Generator). According to the manufacturer's datasheet, the maximum output is a 10 $V_{p\text{-}p}$ sine wave amplitude. This peak-to-peak maximum potential was inserted into the COMSOL model through an electric potential boundary condition, being added to one electrode's upper surface. In contrast to the equivalent surface of the other electrode, a ground boundary condition was added. The model material's electric properties are available in data Table 1. A frequency domain study was performed at 60 kHz, the same frequency used by Brighton et al. to stimulate bone cells with a CCoupled setup. The bioreactor lid design was constructed to accommodate four electrodes, one pair per every two side-by-side scaffolds.

Perfusion system development **(Step 5)**.

A custom-made peristaltic pump system was developed on a low-cost 12 V DC peristaltic pump (PTRobotics, Portugal). This pump is connected to the bioreactor with a Tygon LMT55 3-Stop (Cole-Parmer GmbH, Germany) peristaltic tube with an internal diameter of 2.79 mm. The created perfusion system concept includes options for two different kinds of operation: short-term cell cultures (few days) and long-term cell cultures (weeks to months), Figure S2. Short-term cultures require only one peristaltic pump connecting the bioreactor's outlet and inlet. This setup does not require liquid-level sensors to balance the main chamber liquid content since the amount of liquid that exits the bioreactor's outlet enters at the same rate on its inlet. Any culture medium replacement, if required, will have to be manually performed in this operation mode.

Long-term cell cultures require multiple peristaltic pumps, allowing for independent control of the flow on the inlet and outlet of the developed bioreactor and automatic culture medium renewal without any intervention. In this operation mode, the inlet pump function is to fill the bioreactor. In contrast, the outlet pump is connected to the developed channel network that controls the fluid-induced shear stress at each culture region (scaffold position) along with the mass transport that the cell culture will experience. The coordinated work of the two pumps, managed by the liquid level sensor FIT0212 (DFRobot) reading, is responsible for alternating the inlet and outlet pump's flow rate, maintaining a proper culture medium circulation and a stable culture medium volume. An electronic control circuit was developed to control the pump rotation per minute (RPM) with a microcontroller unit (MCU) output. The developed circuit schematic is shown in Figure S3 and allows the MCU to control a transistor output power (TIP120) with an 8-bit PWM signal, where the number of pulses relates to the pump debit flow rate, as shown in Figure S4. The pump debit was obtained experimentally by measuring the amount of liquid moved in one minute of operation for multiple PWM possible values. According to the selected operation mode, the peristaltic pumps were mounted into a specially developed support platform 3D printed with an Ender 3 S1 Pro 3D FFF printer (Creality, China) using C8 material (3D4Makers, Netherlands). The printer specifications were set accordingly with the C8 manufacturer datasheet (printing temperature: 210°C, bed temperature: 50°C, maximum printing speed: 35 mm/s). The support platform CAD file and the 3D printed 3MF file are available in the open source archive (https://doi.org/10.5281/zenodo.7695700), with all the information required for successfully reproducing the developed peristaltic pump support.

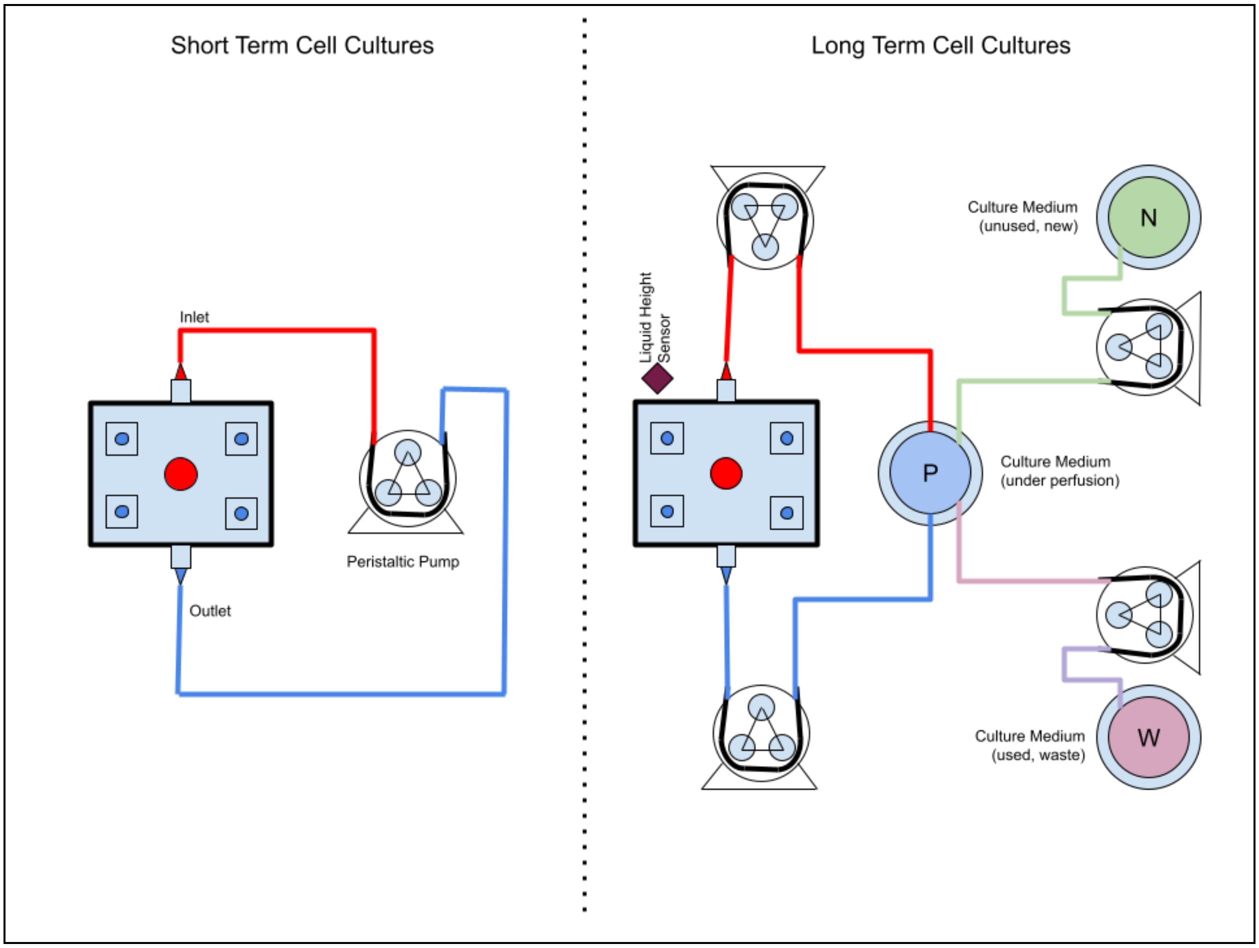

Figure S2 - Top-view schematic of the developed perfusion system modes of operation and their required experimental setups.

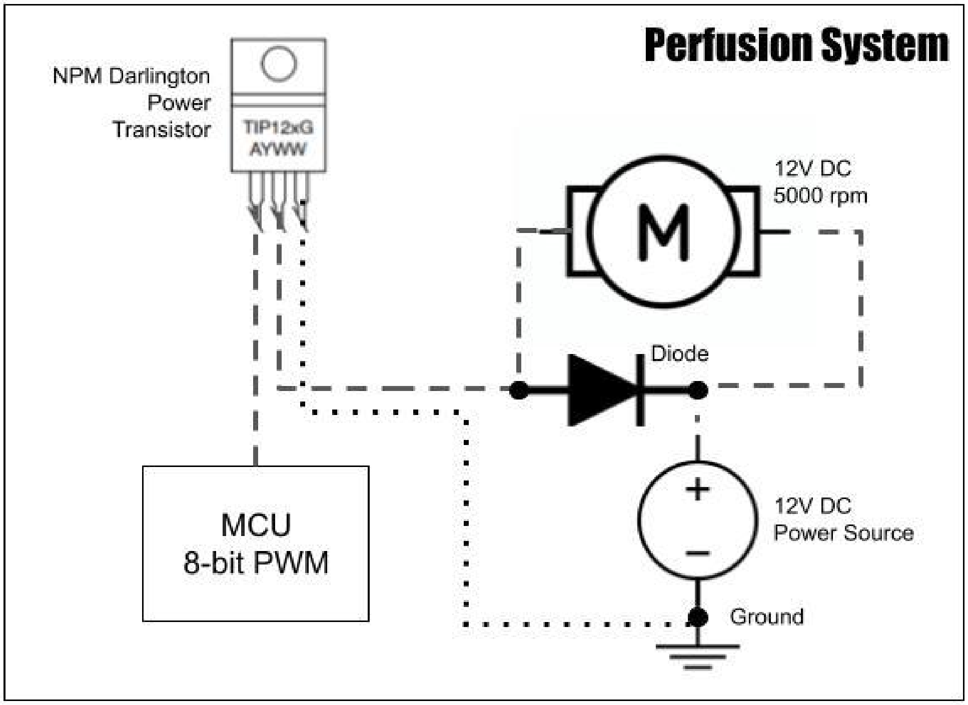

Figure S3 - Circuit schematic of the perfusion system developed for one peristaltic pump motor controlled by an MCU.

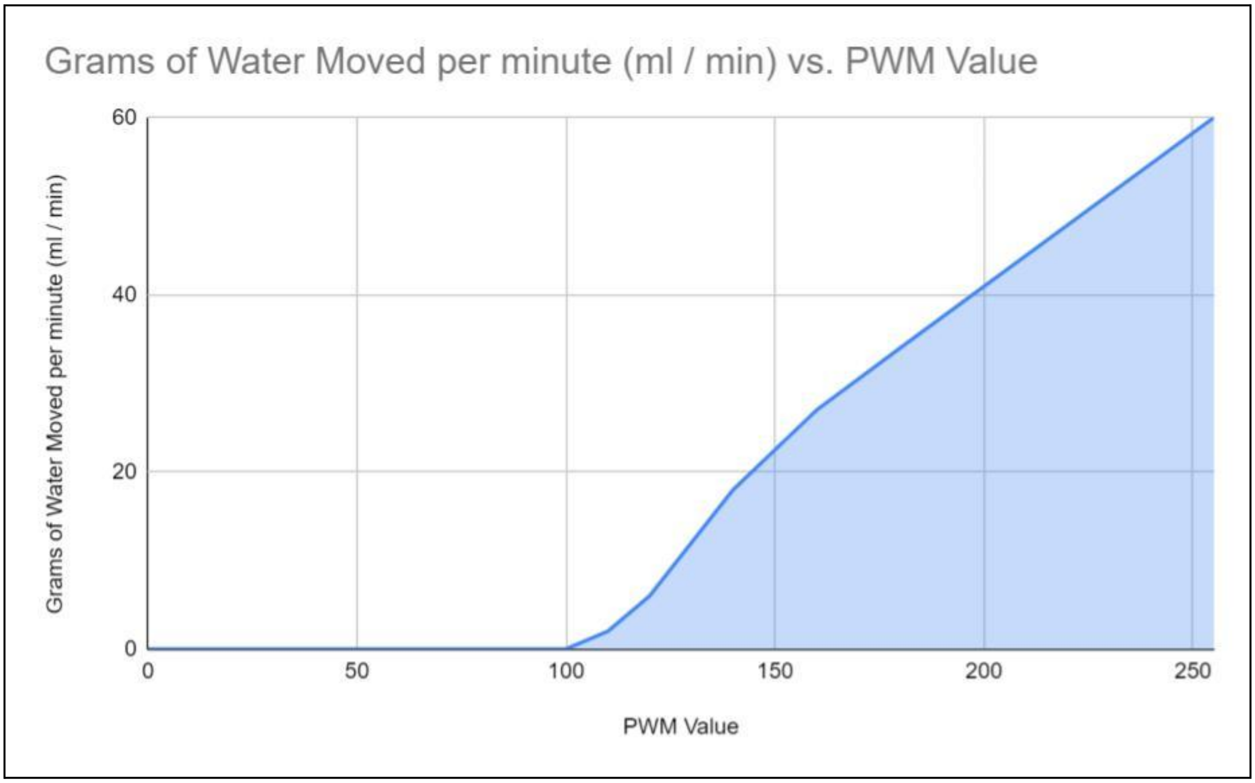

Figure S4 - Plot showing the experimentally obtained relation between the developed peristaltic pump volume debit versus the MCU 8-bit PWM signal output.

Culture medium heater **(Step 6)** and gas mixture control systems **(Step 7)**.

The current bioreactor version still lacks a custom-developed gas mixture control system. An available commercial lab CO2 incubator (MCO-19AIC, SANYO Electric Co, Japan) set the CO2 and O2 mixture for in vitro cell culture validation, surpassing the absence of a custom-developed gas mixture system. The temperature was also set to 37°C in the same incubator system. Several hypotheses for this future development update may include Peltier plates as a heating element and a closed-loop air mixture system with permeable gas membranes (link1) to perform cell culture oxygenation.
Link1 - (https://www.permselect.com/markets/bioreactor)

Sensor integration (pH, dissolved O2, temperature) and microcontroller unit firmware development **(Step 8)**.

The MCU selected for this bioreactor development was the Arduino Nano RP2040 Connect (Arduino, Italy) board, due to being rooted in an open-source electronics platform while maintaining high processing power capabilities (Raspberry Pi® RP2040, dual-core Arm Cortex M0+ running at 133 MHz, 264 KB of SRAM, 16 MB of flash memory) and extensive connectivity options (Wi-Fi and Bluetooth®). The firmware with all bioreactor operations was created in Arduino IDE (version 1.8.19) with the Arduino programming language. The complete developed firmware code file for the selected Arduino board and the correspondent global purpose input-output (GPIO) connection schematics are available in Figure S5. A galvanic dissolved oxygen probe (ENV-40-DO, Atlas Scientific) was connected to the Arduino through the supplied electrically isolated EZO™ carrier board according to the manufacturer's instructions under the UART communication protocol. A pH probe, Gravity: Analog pH Sensor/Meter Kit V2 and its adapter board were connected to the MCU through an analog GPIO pin connected to a multichannel 10-bit analog-to-digital converter. Two thermistors (NTC 3950) were added, both with an individual voltage divider circuit (see Figure S6), to allow the determination of the temperature-dependent resistance value by using the known reference potential of 3.257 V and a resistor with one kOhm. The thermistor's potential reading was acquired by an analog pin using a wire-up identical to the pH probe reading. For data logging during the bioreactor operation, a MicroSD card adapter was added so that each sensor output and other operational data (e.g., perfusion pump power, electric stimulation turning on/off) with their timestamp are continuously recorded and can be further analyzed at the end of the cell culture process. A white LED with a current-limiting resistor was added for debugging purposes. Also, a voltage regulator (LM317t) was integrated into the system to convert the 12 V power source to 5 V (defined by two resistors, R1: 120 ohms, R2: 360 ohms), obtaining the required potential source to power both sensor probes and the card adapter circuit board.

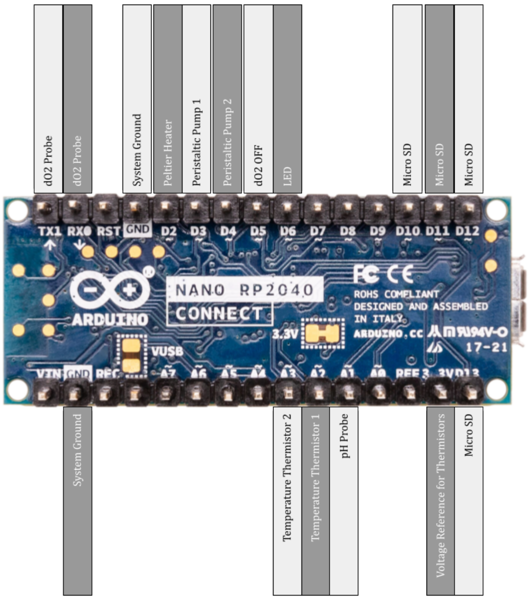

Figure S5 - MCU GPIOs routing for the developed bioreactor, considering the selected board (Arduino Nano RP2040 Connect).

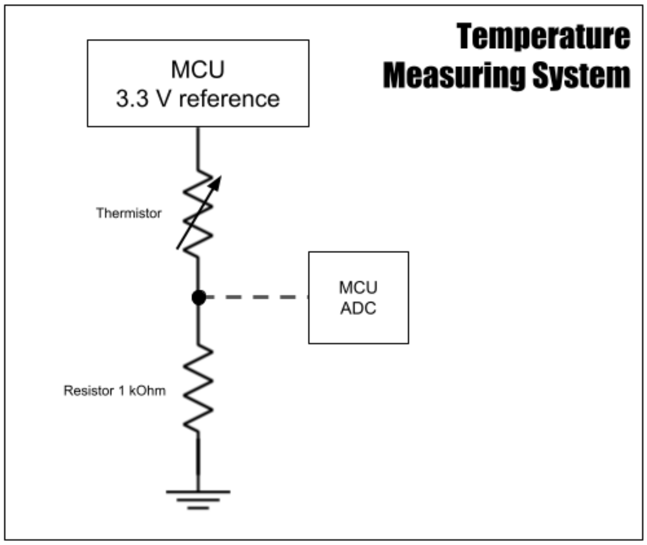

Figure S6 - Circuit schematic developed to obtain thermistor temperature readings by an MCU.

Bioreactor and scaffold selected designs production—integration of developed parts into a remote-controlled cell culture-ready system **(Step 9)**.

The developed bioreactor's final design physical structures were 3D printed with a commercial fuse filament fabrication (FFF) 3D printer (Ender 3 S1-Pro, firmware version 2.0.8.15F4, Creality). Designs were exported in STL file format from the CAD environment and post-processed for 3D printing in the Ultimaker Cura slicer (version 5.1.1, Ultimaker BV) from where the 3MF and GCode files were obtained. This process was applied to all designed parts, including the improved parts that are the outcome of the design decisions supported by numerical models. All those parts were 3D printed with C8 composite material (3D4Makers, Netherlands), known for its excellent layer adhesion and surface quality. Our group previously tested this material for biocompatibility, presenting good results in *in vitro* cytotoxicity tests following the ISO 10993-5 and ISO 10993-12 guidelines [SM1]. All C8 parts were glued and isolated, respectively, with medical adhesive silicone (Sylastic™ type A, Dow Corning) and polydimethylsiloxane (PDMS, Sylgard 184 Silicone Elastomer Kit, applied in a 10:1 (w/w) ratio of base to curing agent) and left to dry overnight. The developed electronic systems (Table S3) were assembled into the same circuit breadboard to test each system individually or in a combined manner. A Bluetooth communication protocol (Bluetooth Low Energy - BLE) was implemented within the Arduino RP2040 MCU to send commands activating/deactivating the developed bioreactor systems and to transfer data from the sensors in real time. This control can be performed directly using a free BLE scanning application (nRF Connect, Nordic Semiconductor ASA) or any custom-developed application that implements the BLE communication protocol following the service and characteristics provided in Table S2. To this end, a simple application was built with the free, cloud-based service MIT App Inventor platform. The developed App was debugged with the MIT AI2 Companion App application in an Android OS environment configured as a BLE client. Its user interface (Figure S7) allows one to write to or read from the implemented BLE service characteristics. The developed bioreactor design with all its composed parts, circuits, firmware, and software is licensed and distributed under the Creative Commons: Attribution-ShareAlike 4.0 International (CC BY-SA 4.0, https://creativecommons.org/licenses/by-sa/4.0/) and is available for download in a public server (https://doi.org/10.5281/zenodo.7695700). Twelve scaffolds were produced for the selected scaffold hypothesis with better numerical predictive outcomes for osteogenesis, considering the combined influence of fluid-induced shear stress and electric field stimulation. Their production was performed with the same FFF 3D printer used for the bioreactor but with polycaprolactone - PCL material (Facilan PCL100, 3D4Makers, Netherlands). PCL was printed accordingly with the manufacturer's advised settings (printing temperature: 135°C, bed temperature: 30°C, maximum printing speed: 3 mm/s). A G-code file to 3D print the selected scaffold geometry was implemented with a custom-developed Python routine that is made available (https://doi.org/10.5281/zenodo.7695700).

SM1 - Meneses, João, João C Silva, Sofia R Fernandes, Abhishek Datta, Frederico Castelo Ferreira, Carla Moura, Sandra Amado, Nuno Alves, and Paula Pascoal-Faria. 2020. "A Multimodal Stimulation Cell Culture Bioreactor for Tissue Engineering: A Numerical Modelling Approach." Polymers 12 (4). https://doi.org/10.3390/polym12040940.

Table S2 - List of Bluetooth low energy (BLE) implemented service and their characteristics, attributed UUID, and description. The root UUID was created with a free online tool: (www.uuidgenerator.net).

BLEService	*00000000-2d0e-4151-b5a0-a982ae345882*		Main BLE Service
BLEIntCharacteristic	*12120000-2d0e-4151-b5a0-a982ae345882*	R/W	Blinking Led
BLEIntCharacteristic	*42420000-2d0e-4151-b5a0-a982ae345882*	R/W	Outlet Peristaltic Pump
BLEIntCharacteristic	*42420001-2d0e-4151-b5a0-a982ae345882*	R/W	Inlet Peristaltic Pump
BLEIntCharacteristic	*64640000-2d0e-4151-b5a0-a982ae345882*	R/W	Peltier Heater
BLEIntCharacteristic	*64640001-2d0e-4151-b5a0-a982ae345882*	R/W	Peltier Calibration Routine
BLEFloatCharacteristic	*71710000-2d0e-4151-b5a0-a982ae345882*	R/N	NTC Temperature Sensor 1
BLEFloatCharacteristic	*71710001-2d0e-4151-b5a0-a982ae345882*	R/N	NTC Temperature Sensor 2
BLEFloatCharacteristic	*71710002-2d0e-4151-b5a0-a982ae345882*	R/N	dO2 Probe Sensor
BLEFloatCharacteristic	*71710003-2d0e-4151-b5a0-a982ae345882*	R/N	pH Probe Sensor

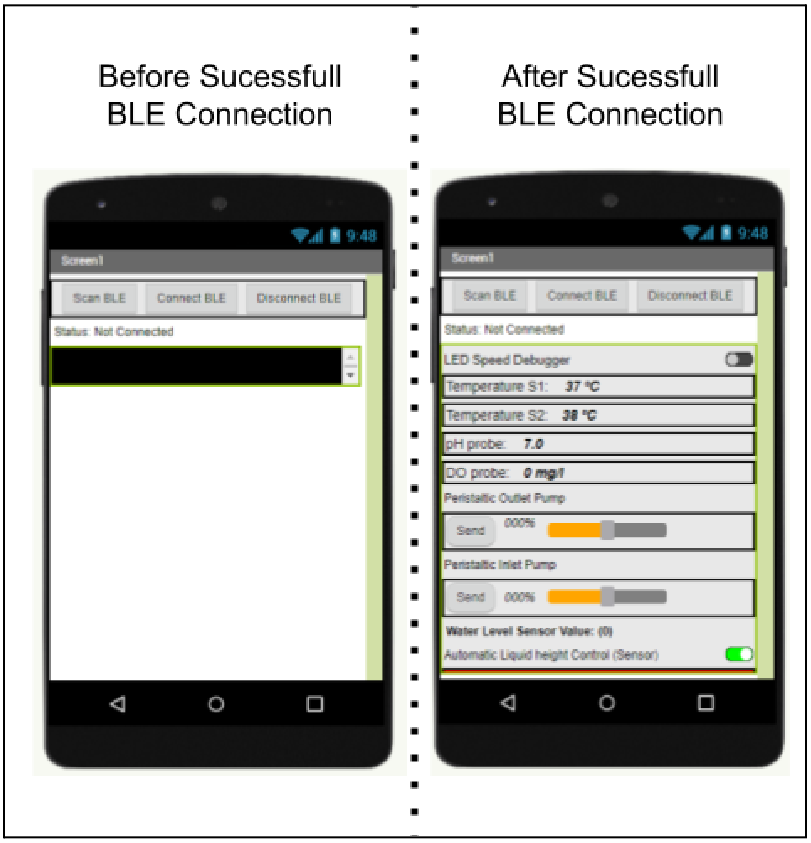

Figure S7 - Custom-developed user interface (UI) to control and communicate with the bioreactor. Build for Android OS with MIT App Inventor platform.

Table S3 - Required bioreactor components (electronics and material), their reference, and price in euros grouped by each bioreactor developed system. Jumper cables, development breadboards, screws, or other connections are not considered. The electric field signal generator acquisition, 3D printer acquisition, and operator spent hours were also not considered for the costs. All electronic components were obtained from botnroll.com (Portugal) and ptrobotics.com (Portugal).

Peristaltic Pump System
(System Cost: 78,77 €)

- 12 V Peristaltic Pump (PTR009795 / PTRobotics, Portugal) - 2x - 40,60€
- C8 3D printed dual peristaltic pump support (DIY) - 1x - 3€ (estimative)
- TIP120 NPN Power Darlington - 2x - 1,10€
- 1N4007 Diode - 2x - 0.12€
- Peristaltic Tygon Tube (TYG LMT55 ID 2.79 3-STOP) - 4x - 7,50€ (estimative)
- Power Supply TracoPower TXM 015-112 - 1x - 25,77€

Heater System
(System Cost: 94,05 €)

- 12 V Peltier plate (TEC12705, 57W, 40x40 mm) - 1x - 16,05€
- Titanium 3D printed heatsink (DIY) - 1x - 40,00€ (estimative)
- C8 3D printed channels, hoses and structures (DIY) - 1x - 10,00€
- Sylgard 184 PDMS (used as glue) - 1x - 10,00€ (estimative)
- Power source ORNO 12V 3A - 1x - 18,00€

Sensing and Controller Systems
(System Cost: 498,71 €)

- Arduino Nano RP2040 Connect - 1x - 25,20€
- pH probe (Gravity: Analog pH Sensor/Meter Kit V2) - 1x - 44,95 €
- Dissolved O2 Probe Kit (ENV-40-DO, Atlas Scientific) - 1x - 399,00€
- Thermistor (NTC 3950 10KΩ 1%) - 2x - 3,80€
- 1 kOhm resistor - 2x -0.04€
- MicroSD Card Adapter - 1x - 3,65€
- Cartão Kingston Canvas 32GB Select Plus MicroSDHC UHS-I A1 (Class 10) - 1x - 5,80€
- Superbright white led, 3mm - 1x - 0.12€
- Resistor 220 Ohm - 1x - 0.05€
- LM317T Adjustable Voltage Regulator - 1x - 0,60€
- Liquid Level Sensor FIT0212 (DFRobot) - 1x - 15,50€

CCouple Electrical Stimulation System
(System Cost: 54,96 €)

- ITOPET film 100x200 mm - 43.96 €
- Parts 3D printing in C8 material - 4x - 1,00 €
- Sylgard 184 PDMS (used as glue) - 1x - 10,00€ (estimative)

Main Culture Chamber, Lid, Holders, Connectors
(System Cost: 16,53 €)

- Parts 3D printing in C8 material - 8 parts - 6.53 €
- Sylgard 184 PDMS (used as glue) - 1x - 10,00€ (estimative)

TOTAL COST PER BIOREACTOR: 743,02 €

Bioreactor systems and scaffold validation pre-culture **(Step 10)**.
All developed electronic systems were tested individually to guarantee proper functioning. With every system performing as expected, a series of pre-culture validations were then conducted as described below.

Watertight test. The developed bioreactor main chamber was flooded first with distilled water and afterward with basal DMEM culture media for 24h at 37°C in an incubator (Venticell 22 Eco Line, MMM Group). Below the bioreactor, a dry paper was placed to observe possible external leakages. Also, the liquid height inside the bioreactor was marked to find possible inner leakages (from the culture chamber to empty 3D printing infill space).

Perfusion velocity measurement test. The empty bioreactor culture chamber containing the selected holders in their position was filled with distilled water. This test was performed while working with two peristaltic pumps. First, the inlet pump was set at 100% power. When the water reached the maximum capacity of the bioreactor, the inlet pump was turned off, and the outlet pump was turned on, working also at 100% power. A video was recorded with a standard cellphone camera and a fluorescent-yellow dye (FT601 Dye, Anderson Manufacturing) that was slowly poured into the chamber using a syringe. The dye flowing to the outlet allowed us to estimate its velocity in the selected holder region by measuring its movement against a millimetric paper pattern (size reference) at the bottom of the bioreactor chamber and by measuring the elapsed time between the recorded video frames (VideoLan with Time v3.2 VLC Extensions). A screen capture was taken when the dye entered the selected holder region and another when the dye reached the selected holder outlet hole. To measure the distance traveled by the dye, we run the video frame-by-frame, analyzing those images with a free online tool (www.imagemeasurement.online). The experimentally obtained fluid velocity was then compared with the CFD numerical model prediction for the same fluid flow conditions in the culture chamber without the scaffold presence to validate the numerical model predictions (S10a, S10b).

24-hour continuous perfusion test. To evaluate if the developed peristaltic pump system can maintain the culture chamber with the same amount of liquid for short-term cell culture (malfunctioning of one of those pumps or the liquid level sensor would flood or empty the main culture chamber). Both peristaltic pumps were connected to the developed bioreactor culture chamber and a liquid reservoir. The capacitive liquid level sensor was connected to the lid in its designed compartment. Both pumps were set at a 100% pump rate, and the system was left operating continuously for 24 hours, alternating its inlet with outlet flow according to the liquid level sensor readings. One pump bioreactor configuration was also tested for the same amount of time.

CCoupled Electrical Stimulation System Test. Two electrodes were mounted 10 mm apart in a specifically designed support filled with basal culture medium (Figure S11) to test the performance of the developed ITO PET capacitive electrodes. An electric potential sine wave with an amplitude of 10 Vpk-pk at 60 kHz was applied (72-3555 Waveform Generator, TENMA), and the resultant electric current was measured (DSOX1102A Oscilloscope, KEYSIGHT) through the potential drop in a known 21.82 kOhm resistor mounted in series with the ground electrode.

An electric field calculator for CCoupled setups (previously developed by our group [SM2]) was employed to obtain the predicted electric current and resultant electric field, considering the wave applied, the material parameters of the entire setup (described in the main manuscript) and an electrode exposure surface of 120 mm^2 (equivalent radius of 6.18 mm). The experimental electric current measurement results were confronted with model prediction to validate the electrical field numerical model.

SM2 - Meneses, João, Sofia Fernandes, Nuno Alves, Paula Pascoal-Faria, and Pedro Cavaleiro Miranda. 2022. "Author Correction: How to Correctly Estimate the Electric Field in Capacitively Coupled Systems for Tissue Engineering: A Comparative Study." Scientific Reports 12 (1): 12522.

Sensors independent test. The temperature sensors were tested against one commercial thermocouple sensor, used as a golden standard (MM420 thermocouple with adapter, Klein Tools). The pH probe was calibrated and tested against two known pH standard solutions, one at pH 4 and the other at pH 7. The dO2 probe was calibrated and tested against two known points: the atmospheric air and the probe manufacturer's zero dissolved oxygen solution.

Scaffold morphology analysis. Micro-computed tomography (µCT) evaluated the scaffold structure for dimensional control using a SkyScan 1174TM (Brucker, Kontich, Belgium). The scaffold was randomly selected from the production, and multiple images were captured at different points (Figure S12). The measurements were performed using ImageJ v.1.53t software in three pores and filaments. For the µCT, the parameters used for the digitalization of the samples were a rotation step of 0.6 degrees around the mediolateral axis, an acceleration voltage of 50 kV, a source current of 800 µA; an exposure time of 6500 ms; a frame average of 3 with pos-processing parameters (smoothing of 1, ring-artifact of 4). Reconstruction was done using the software NRecon (version 1.6.8.0), using the included CTVox program to do the 3D realistic visualization of the scanned samples. µCT analysis allows us to evaluate scaffold characteristics, such as overall geometry comparison with CAD ground truth.

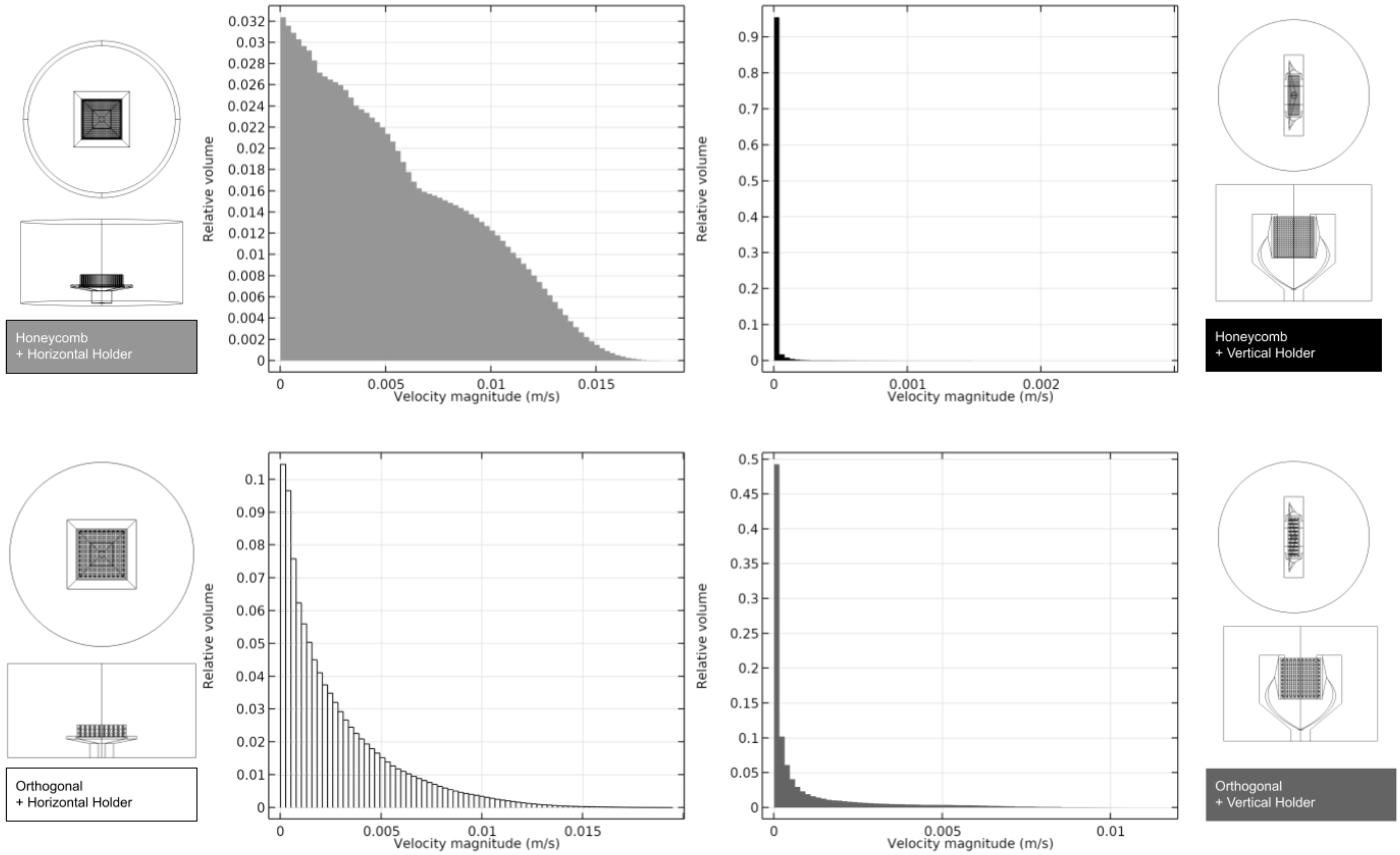

Figure S8 - Relative volumetric distribution of the velocity magnitude (m/s) for all possible combinations of selected scaffolds and holders when the bioreactor outlet is subjected to the maximum peristaltic pump rate (50 ml/min). All data was obtained from CFD numerical model predictions at the scaffold region of interest.

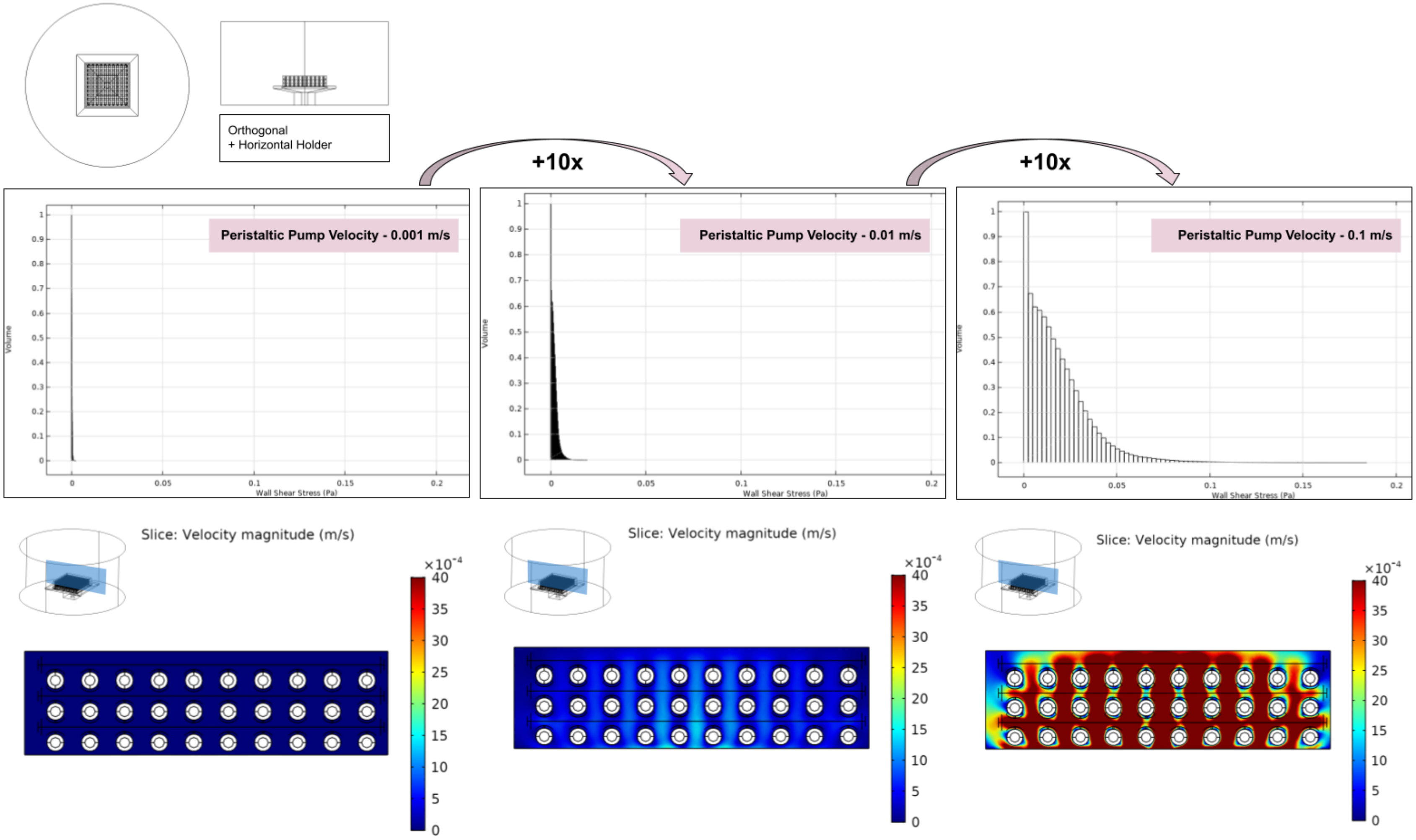

Figure S8a - Comparison between different peristaltic pump flow rates to exemplify how changing this inlet/outlet velocity will, in turn, narrow or expand the range of applied shear stress. This velocity adjustment will allow to tune mechanical stimulation.

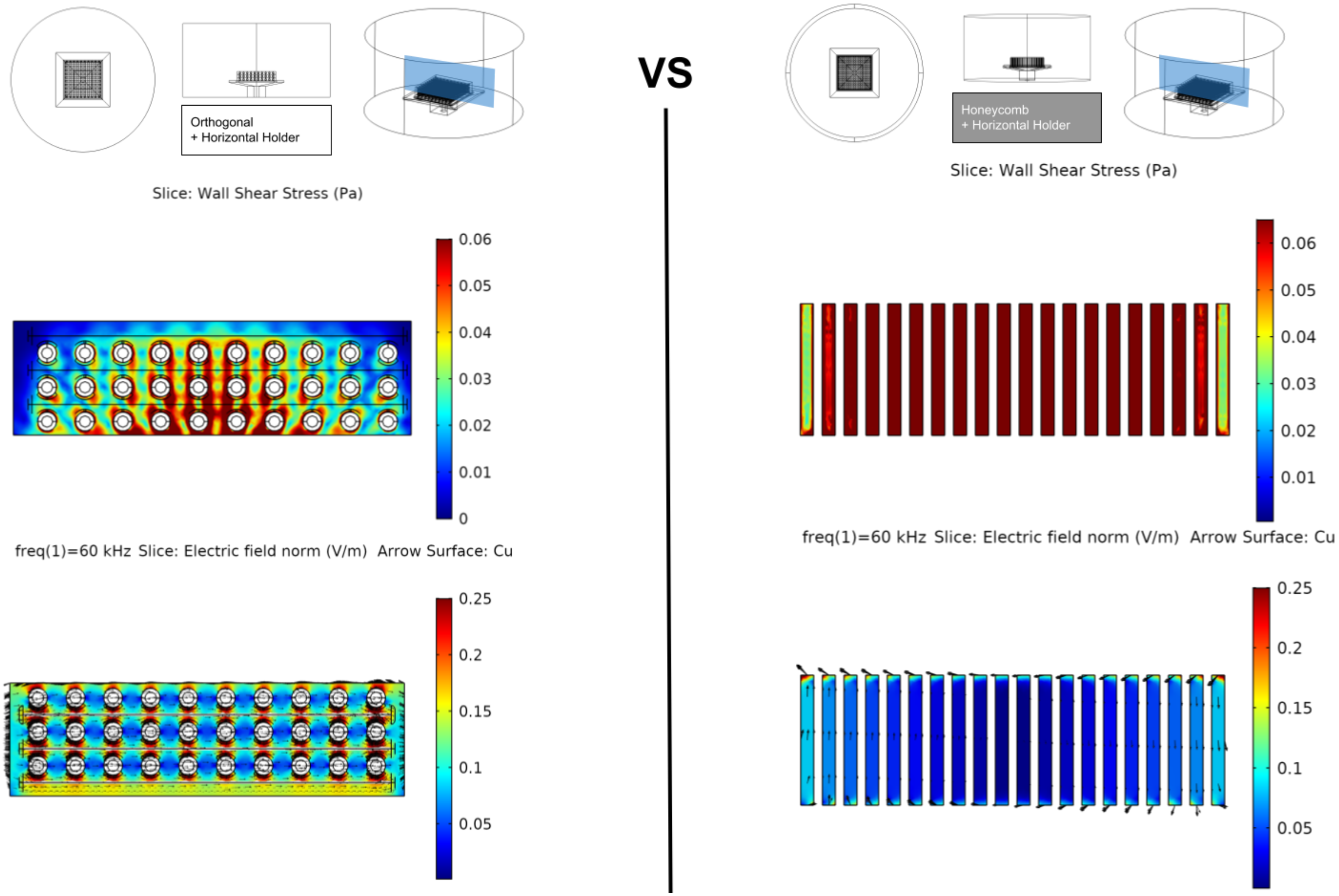

Figure S8b - Comparison between the orthogonal and honeycomb scaffold designs, using a slice as a term of comparison regarding fluid flow-induced shear stress and electric field magnitude. Both scaffold designs are placed in a horizontal holder under the same flow conditions and the same CCoupled electric stimulation protocol.

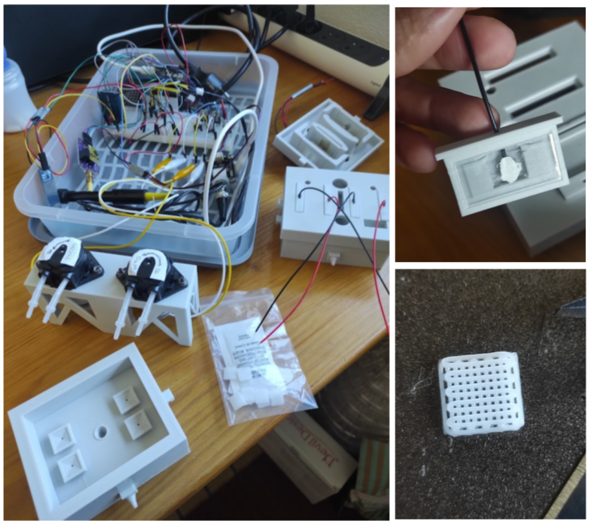

Figure S9 - (a) Complete developed bioreactor with perfusion system, sensors, and control electronics as described; (b) developed ITO PET capacitive electrode; (c) produced PCL orthogonal scaffold;

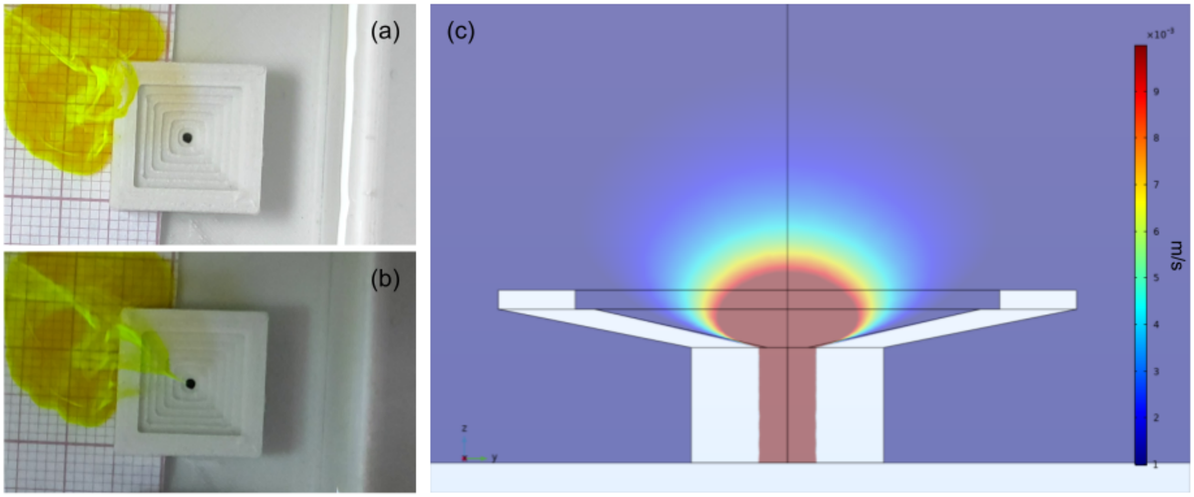

Figure S10a - Fluid flow velocity measurement with dye movement in video frames, comparison with numerical models for the same scaffold holder region. (a) video frame with timestamp 13s042ms; (b) video frame with timestamp 16s005ms; (c) Fluid flow numerical model prediction for the bioreactor scaffold holder region without scaffold.

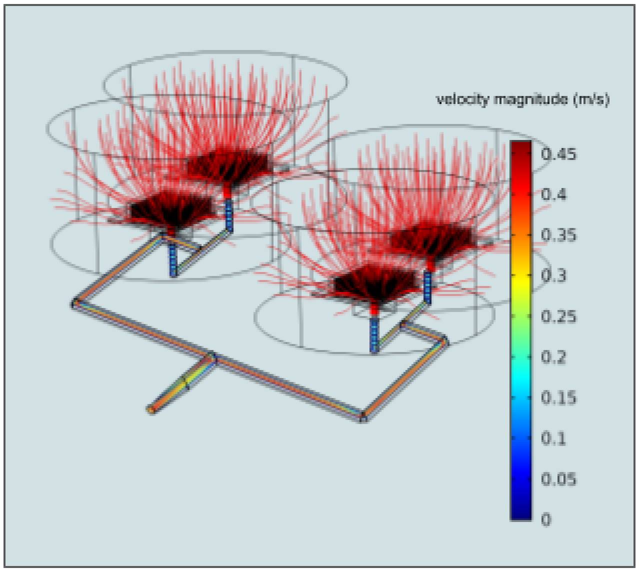

Figure S10b - CFD model predictions. The image contains a fluid flow velocity plot for the channels network model, considering the channel geometry and maximum peristaltic pump capability. Four streamlined result plots were added considering the holder+scaffold model, considering horizontal holders.

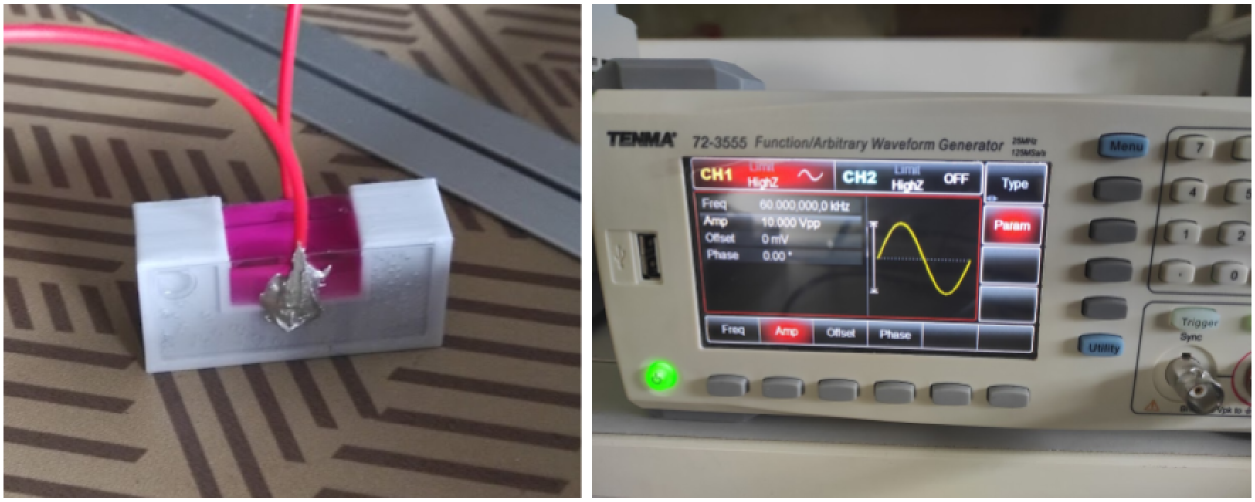

Figure S11 - Left: CCoupled electrical stimulation system test apparatus with two ITO PET electrodes as described. Right: Applied waveform and signal source.

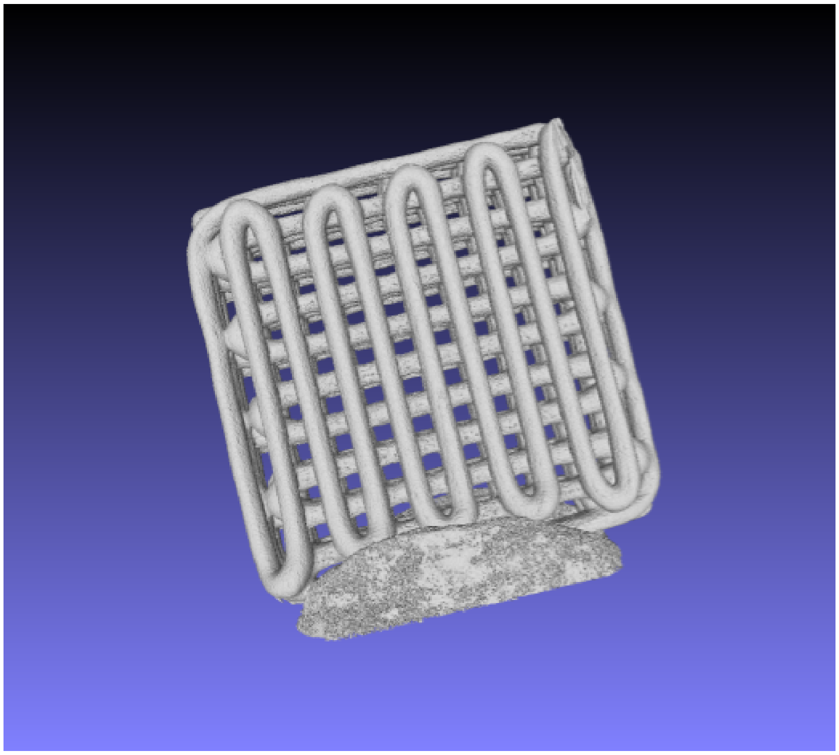

Figure S12 - µCT scan reconstruction of the produced PCL orthogonal scaffold sample (at the bottom is visible the supporting wax required to run the µCT scan).

Bioreactor and scaffold in-vitro cell culture (**Final Step**).

Cell culture and PCL scaffold seeding. Human osteoblast-like MG-63 cells (ATCC® CRL-1427™) were thawed from the nitrogen cell bank available at the Stem Cell Engineering Research Group at the Institute for Bioengineering and Biosciences (iBB)-Instituto Superior Técnico and expanded in culture flasks using high-glucose Dulbecco's Modified Eagle Medium (DMEM, Gibco™, Thermo Fisher Scientific) supplemented with 10% v/v fetal bovine serum (FBS, Gibco™, Thermo Fisher Scientific) and 1% v/v penicillin-streptomycin (pen-strep, Gibco™, Thermo Fisher Scientific) at 37 °C and 5% CO2 in a humidified atmosphere. The cells were passaged to new flasks or seeded in scaffolds when confluence (≈80–90%) was reached. The culture medium was wholly renewed every 2–3 days.

Before the in vitro cell culture experiments, 3D porous PCL scaffolds were sterilized through ethanol 70% washing and by ultraviolet light exposure for 2 hours (each side of the scaffold). Then, PCL scaffolds were rinsed three times with a 1% v/v antibiotic-antimycotic (Anti-Anti, Gibco™) solution prepared in phosphate-buffered saline (PBS, GibcoTM) for 3 h. The scaffolds were incubated in culture media for 1 h at 37°C / 5% CO2 to promote cell attachment. Afterward, MG-63 osteoblasts were seeded onto the scaffolds ($2x10^5$ cells/scaffold), previously placed in a 24-well ultra-low attachment plate (Corning). The scaffolds were then incubated for 2 h without culture media to allow initial cell attachment. Media was added to each scaffold, and the cultures were kept for 12 days at 37°C / 5% CO2 to allow cells to populate the whole scaffold structure. Culture media was fully renewed twice a week.

Bioreactor culture of cell-seeded scaffolds. After 12 days of culture under static conditions in 24-well plates (Figure S13), the scaffolds were transferred to the respective bioreactors (Bioreactor EStim vs. Bioreactor No EStim vs. culture well plate (control) and cultured for 48 h in osteogenic induction medium composed of by DMEM supplemented with 10% FBS and 1% pen-strep, 10mM β-glycerophosphate (Sigma-Aldrich), 10nM dexamethasone (Sigma-Aldrich), and 50 μg/ml ascorbic acid (Sigma-Aldrich). The capacitive electrical stimulation (performed three times for Bioreactor EStim) consisted of 10 V peak-to-peak (5V to -5V), with a frequency of 60KHz for 1 hour per day. After 48 h of culture, 4 cell-seeded scaffolds of each bioreactor condition were collected to analyze cell viability and metabolic activity compared to the control sample (scaffolds cultured in culture-well plates).

Assessment of cell metabolic activity and viability. The metabolic activity of MG-63 osteoblasts in the different experimental scaffold groups was evaluated right before and after Bioreactor culture using the AlamarBlue® assay (Thermo Fisher Scientific) according to the manufacturer's guidelines. Briefly, scaffold samples were incubated with a 10% v/v AlamarBlue solution (prepared in culture media) for 4 h at 37°C/5% CO2. Afterward, the solution fluorescence intensity was measured in a microplate reader (Infinite®200 PRO, TECAN) with an excitation wavelength of 560nm and emission wavelength of 590nm. Four scaffolds (N=4) were considered for each experimental group, and each scaffold sample's fluorescence intensity values were measured in triplicate. PCL scaffolds without cells submitted to the same experimental conditions were used as blank controls.

The viability of MG-63 osteoblasts in the final constructs after bioreactor culture with and without EStim was studied using a LIVE/DEAD kit (Life Technologies). Samples were incubated in PBS containing 2 μM calcein AM (stains the live cells green) and 4 μM ethidium homodimer-1 (stains the dead cells red) for 1 h at room temperature, washed with PBS and immediately imaged under a fluorescence microscope (LEICA DMI3000B, Leica Microsystems).

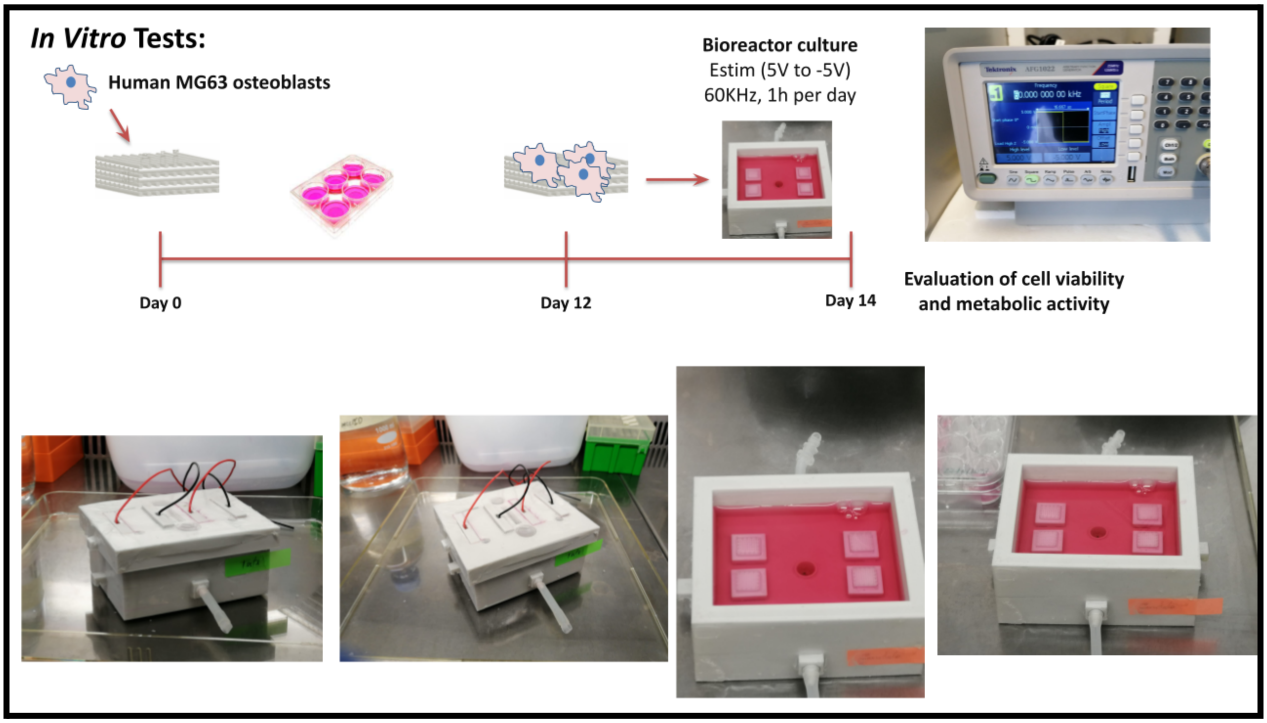

Figure S13 - Cell culture timeline for the preliminary bioreactor in vitro validation tests.

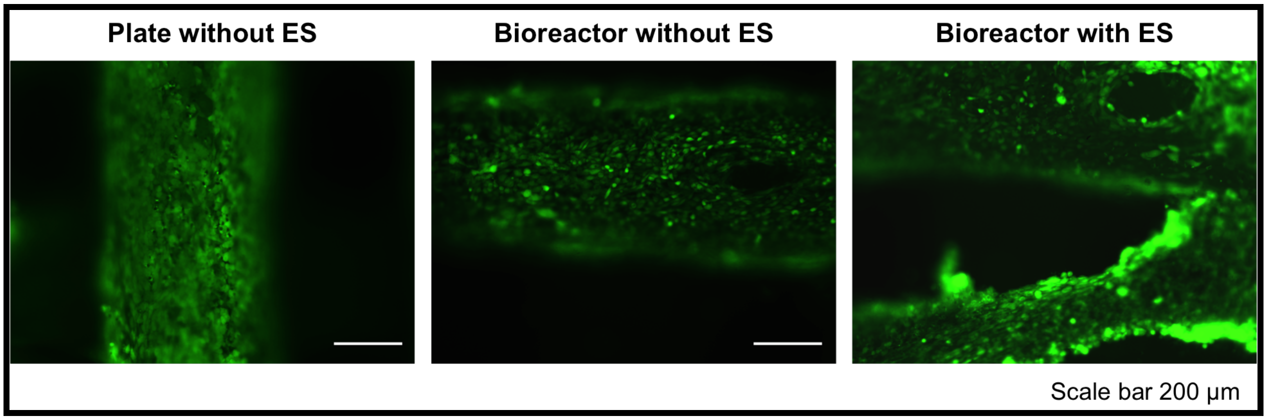

Figure S14 - Results from the bioreactor preliminary human MG-63 osteoblasts cell culture viability tests performed with LIVE/DEAD staining at day 14 (after 3x CCoupled stimulation and 48h bioreactor culture).
